# *Prorocentrum insidiosum* sp. nov. (Prorocentrales, Dinophyceae): Morphological and Phylogenetic Characterization of a Mucosphere Producing Dinoflagellate From the “*cordatum* Group”

**DOI:** 10.1111/jeu.70017

**Published:** 2025-05-30

**Authors:** Michaela E. Larsson, Gustaaf Hallegraeff, Martina A. Doblin, Urban Tillmann

**Affiliations:** ^1^ Department of Water and Environmental Regulation Aquatic Science Branch Joondalup Western Australia Australia; ^2^ Climate Change Cluster (C3) University of Technology Sydney Broadway New South Wales Australia; ^3^ Institute for Marine and Antarctic Studies University of Tasmania Hobart Tasmania Australia; ^4^ Sydney Institute of Marine Science Mosman New South Wales Australia; ^5^ Alfred Wegener Institute, Helmholtz Center for Polar and Marine Research Bremerhaven Germany

**Keywords:** biological carbon pump, mixoplankton, morphology, mucosphere, mucus trap, phagotrophy, phylogeny, plankton, taxonomy

## Abstract

*Prorocentrum* cf. *balticum* was the provisional designation assigned to strains of a small, pelagic, mixoplanktonic dinoflagellate found to produce carbon‐rich mucilage‐based prey capture devices, termed “mucospheres.” Here we characterize the morphology and phylogeny of the strains, describe them as *Prorocentrum insidiosum* sp. nov., and discuss common morphological features among the six species of the phylogenetically defined *P. cordatum* group. Cells of *P. insidiosum* sp. nov. were round to slightly ovate in lateral view, 12–16 μm long and 8–15 μm deep, and laterally compressed. Scanning electron microscopy revealed the thecal plates were densely ornamented with short spines and there were two size classes of pores irregularly distributed across both plates, and a row of two to four large round pores in apical‐ventral position on the right thecal plate. The periflagellar area consisted of eight platelets, and there were two prominent wing‐like apical projections in the form of a double layered curved structure on platelet 1 with additional projections on most other platelets except platelet 4. *Prorocentrum insidiosum* sp. nov. is distinct from all genetically represented species within the genus and possesses a unique combination of morphological features differentiating it from other protologues of small *Prorocentrum* species.

**Abbreviations**
ap: accessory pore
bp: base pairs
CCMP: The Provasoli‐Guillard National Center for Culture of Marine Phytoplankton
DAPI: 4′,6‐diamidino‐2‐phenylindole
DIC: Differential Interference Contrast
em: emission
EtOH: ethanol
ex: excitation
FITC: fluorescein isothiocyanate
fp: flagellar pore
GTR: general time‐reversible
GTR + G: general time‐reversible with gamma
HMDS: hexamethyldisilazane
IMOS: Integrated Marine Observing System
ITS: internal transcribed spacer
LM: light microscopy
LSU: large subunit
MAFFT: Multiple Alignment using Fast Fourier Transform
ML: Maximum Likelihood
MUSCLE: MUltiple Sequence Comparison by Log‐Expectation
NCBI: National Center for Biotechnology Information
NCMA: National Center for Marine Algae and Microbiota
PHYML: PHYlogenetic inferences using Maximum Likelihood
RCC: Roscoff Culture Collection
SD: standard deviation
SEM: scanning electron microscopy
SSU: small subunit

## 1 Introduction

**FIGURE 1:**
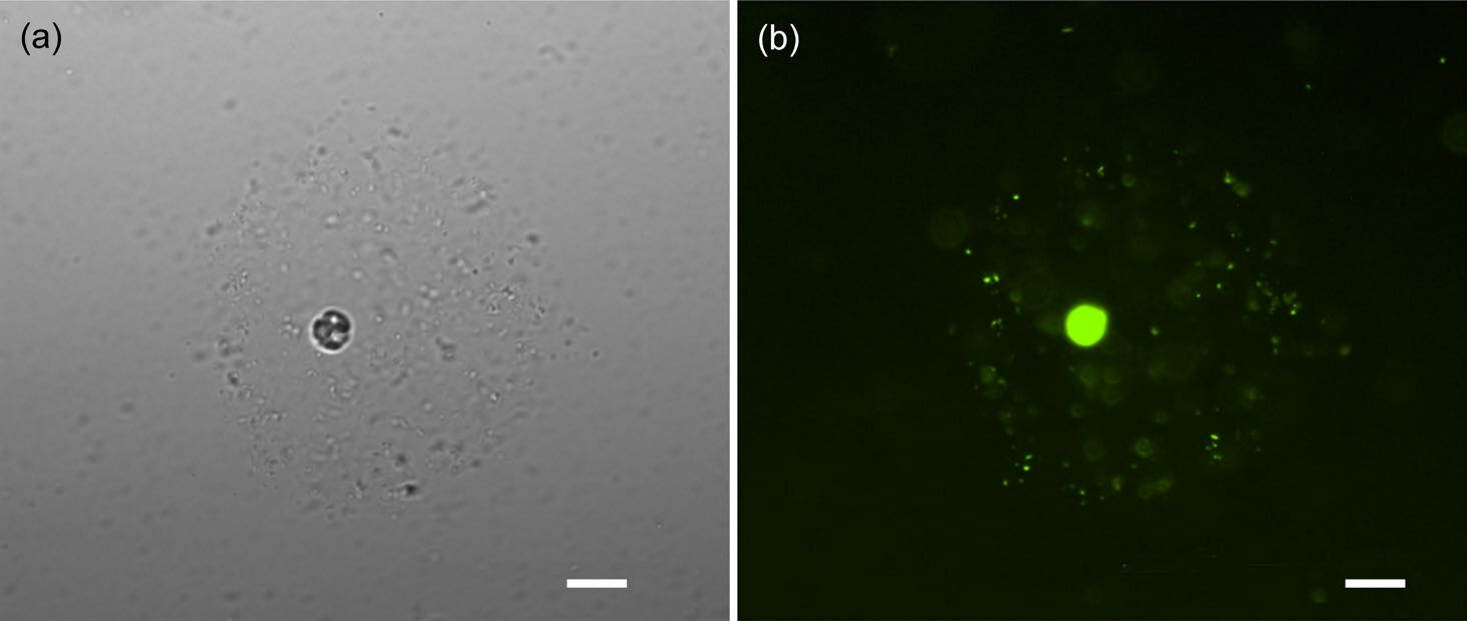
(a, b) Image of a *Prorocentrum insidiosum* sp. nov. cell with its mucosphere and immobilized prokaryotes visualized under brightfield (a) and using a FITC 480/30 nm ex 535/45 nm em filter on a fluorescence microscope after addition of the fluorescent stain SYBR Green (b). Scale bars = 20 μm.

Some species from the dinoflagellate genus *Prorocentrum* Ehrenberg (Dinophyceae) are known to construct mucilage‐based prey capture devices. The construction and use of such “mucospheres” was first observed in strains from Australia provisionally denominated as *Prorocentrum* cf. *balticum* by Larsson et al. (2022), who also demonstrated the potential for these carbon‐rich structures to disproportionally contribute to the vertical flux of carbon in the ocean, influencing the Biological Carbon Pump. Larsson et al. (2022) used multiple lines of evidence to show that *P*. cf. *balticum* likely has a broad distribution in the ocean and attributed its success in part to its diverse range of metabolic strategies and behaviors. The species was found to be an obligate phototroph, peduncular feeding facultative phago‐heterotroph, constitutive mixotroph as defined by Mitra et al. (2016). The species has its own chloroplasts which it uses for photosynthesis and relies on this to acquire carbon but can also supplement its nutrition through consumption of microbes via a short tubular appendage known as a peduncle. This was the first report of peduncular feeding in a species of *Prorocentrum*, although a peduncle had been detected previously through ultrastructural analysis and this feeding mechanism was suspected for other species within the genus (Schnepf and Winter 1990; Faust 1997). Cells of the Australian strains use mucospheres to attract, capture, and immobilize microbial prey, aiding peduncular consumption and increasing encounter rates (Figure 1). The mucus structures are then abandoned and sink, contributing to the vertical flux of carbon in the ocean. Construction of such “mucus traps” have now also been observed in two other small, pelagic *Prorocentrum* species: the recently described *P. pervagatum* Tillmann, Hoppenrath & Gottschling and the common, well‐studied *P. cordatum* (Ostenfeld) J.D.Dodge (Tillmann, Mitra, et al. 2023) (often considered a senior synonym of *P. minimum* (Pavillard) J. Schiller) (Velikova and Larsen 1999).

The thecate dinophyte genus *Prorocentrum* consists of more than 80 species that are predominately marine or estuarine, have pelagic or benthic habits (Dodge and Bibby 1973; Guiry 2010; Hoppenrath et al. 2013) and can either be photo‐ or mixotrophic (Hansen and Tillmann 2020; Mitra et al. 2023). Many species have a cosmopolitan distribution, are common members of the plankton community, and can form high biomass blooms in coastal habitats (Heil et al. 2005; Ajani et al. 2018). Cells of *Prorocentrum* have a peculiar morphology in that they have only two major thecal plates joined by a distinct sagittal suture. Lacking a cingulum and sulcus, the two flagella instead emerge from the apical flagellar pore (desmokont flagellation) (Dodge 1975). Among the pelagic group, there are several small (< 20 μm) and roundish (in lateral view) species which are particularly challenging to identify because of their size, and most of the older species' descriptions lack ultrastructural details and physical type material. Consequently, there has been considerable taxonomic confusion and ambiguity in the genus.

Historically, *Prorocentrum* species determinations were based on the size and shape of cells; the size, number, and distribution of thecal pores; details of the surface ornamentation of thecal plates; and the presence or absence of conspicuous apical projections (Dodge 1975). With new species determinations, it is now standard to also provide details of the apical periflagellar platelet pattern and complete phylogenetic analysis. Thus, a number of thoroughly examined species have now been described, including *P. nux* Puigserver & Zingone (Puigserver and Zingone 2002), *P. pervagatum* (Tillmann, Wietkamp, et al. 2023) (=*P. criophilum* Gourvil & Gutiérrez‐Rodríguez) (Gómez et al. 2023), *P. thermophilum* F. Gómez, Tangcheng Li, Hu. Zhang & Senjie Lin (Gómez et al. 2023) and *P. spinulentum* Tillmann, Gottschling & Hoppenrath (Tillmann, Gottschling, et al. 2023), along with a re‐examination of a number of other species (Gómez et al. 2021). This has provided some clarity and a solid foundation for further taxonomic studies on this important group of planktonic dinoflagellates.

Here we present a detailed morphological and phylogenetic assessment of the Australian *Prorocentrum* strains from Larsson et al. (2022) and describe them as *Prorocentrum insidiosum* sp. nov. The description is based on a strain originally isolated from the continental shelf waters of the southwest Pacific Ocean, off the coast of Port Hacking in New South Wales, Australia, and includes high‐resolution light and electron microscope imagery, with description of the apical periflagellar platelet pattern, coupled with phylogenetic analysis of individual nuclear rRNA gene regions and concatenated sequences. With *P. insidiosum* sp. nov as a member of the phylogenetically defined “*cordatum* group” we also discuss the common morphological features among the six species that comprise this group: *P. cordatum* (senior synonym of *P. minimum* ), *P. pervagatum*, *P. thermophilum* , *P. spinulentum*, *P. shikokuense* Y.Hada, and *P. insidiosum* sp. nov.

## 2 Methods

### 2.1 Sample Collection, Cell Isolation and Culture Maintenance

Detailed methods describing the sample collection, cell isolation, and culture maintenance of the *P. insidiosum* sp. nov. strains are available in Larsson et al. (2022). Briefly, a water sample was collected from the Port Hacking 100 m Australian Integrated Marine Observing System (IMOS) National Reference Station located on the continental shelf of Southeast Australia (34.120° S, 151.224° E) in September 2018 using a 20 μm plankton net. The collected material was enclosed in a sealed jar and stored in an incubator maintained at 20°C, under ~150 μmol m^−2^ s^−1^ light on a 14:10 h light:dark cycle for 4 weeks with no added nutrients.

Single cells from the *Prorocentrum* genus were isolated using the micropipette technique (Andersen and Kawachi 2005) and placed into individual wells of a 96 multi‐well plate (Falcon, Corning, New York, USA) containing 200 μL, 0.2 μm filter sterilized and autoclaved natural seawater collected from the sampling location, and were incubated under the same conditions. K medium (‐Si) (Keller et al. 1987) was gradually introduced as the cells began to divide.

Four strains of *P. insidiosum* sp. nov. were established (strain codes UTSPH2D1, UTSPH2D4, UTSPH3C1, and UTSPH3D3) and were maintained in 25 cm^2^ (50 mL) sterile vented polystyrene tissue culture flasks (Falcon, Corning, New York, USA) in K medium (‐Si) made from filter sterilized and autoclaved natural seawater collected from the isolation location, at a temperature of 20°C, salinity of 35, under ~150 μmol m^−2^ s^−1^ light on a 14:10 light:dark cycle in a plant growth chamber (Fitoclima S600, Aralab, Rio de Mouro, Portugal).

Additional morphological observations were performed with one strain of *P. cordatum* (1‐B3) isolated from the English Channel (50°14.388′ N, 0°57.366′ E) in July 2018 onboard the research vessel “Heincke.” This strain was established by single cell isolation using the micropipe technique and was grown in K medium (‐Si) (Keller et al. 1987) slightly modified by replacing the organic phosphorous source with 3.62 μM Na_2_HPO_4_. The medium was made with filter sterilized and autoclaved natural seawater collected from the North Sea at a temperature of 15°C, salinity of 32, under ~80 μmol m^−2^ s^−1^ light on a 16:8 light:dark cycle in a plant growth chamber. In addition, for a thorough morphological comparison of *P. insidiosum* sp. nov. with the closely related *P. thermophilum* , two strains of *P. thermophilum* were obtained from the CCMP culture collection (CCMP1787 and CCMP1260) and were grown under the same conditions as described for the *P. cordatum* strain 1‐B3.

### 2.2 Light and Epifluorescence Microscopy

Live and formaldehyde (1% final concentration) or neutral Lugols (1% final concentration) preserved cells were visualized using a compound microscope (Axioskop 2, Zeiss; Jena, Germany) equipped with epifluorescence and Differential Interference Contrast (DIC) optics and a digital camera (Axiocam MRc5, Zeiss). Videos were recorded using a digital camera (Gryphax Jenoptik; Jena, Germany) at full‐HD resolution, and single frame micrographs were then extracted using Corel Video Studio software (Version X8, Coral; Ottawa, Canada). An inverted microscope (Nikon Eclipse Ti, Japan) fitted with FITC 480/30 nm ex 535/45 nm em and a digital monochrome camera (Nikon DS‐QiMc) was also used, and images were captured using proprietary software (NIS Elements v4.60).

The dinoflagellate and its mucosphere were observed under epifluorescence after staining live cultures with SYBR Green I 10,000× (5:100,000 dilution) as per Larsson et al. (2022). Thecal plates were examined using epifluorescence microscopy after cells had been stained with either calcofluor white (Fritz and Triemer 1985) or Solophenyl Flavine (Chomérat et al. 2017). The shape and location of the nucleus were determined after formaldehyde‐preserved cells had been stained with 4′‐6‐diamidino‐2‐phenylindole (DAPI, 0.1 μg mL^−1^ final concentration) for 10 min.

### 2.3 Scanning Electron Microscopy (SEM)

Scanning Electron Microscopy (SEM) was completed for a strain of *P. insidiosum* sp. nov. (UTSPH3D3), both CCMP strains of *P. thermophilum* (CCMP1260 and CCMP1787), and the *P. cordatum* strain 1‐B3.

Cells from a 15 mL aliquot of each culture were concentrated by centrifugation (3220 *g* for 10 min; 5810R, Eppendorf) and preserved with formaldehyde (1% final concentration). For SEM preparation, cells of the formaldehyde‐preserved sample were collected on polycarbonate filters (25 mm diameter, 3 μm pore‐size, Millipore Merck; Darmstadt, Germany) and washed a total of eight times with 2 mL MilliQ‐deionized water, followed by a dehydration series of ethanol (EtOH) at 30%, 50%, 70%, 80%, 95%, and 100%; for 10 min each. Filters were then chemically dried using hexamethyldisilazane (HMDS), first with a 1:1 mixture of HMDS:EtOH, followed by two applications of 100% HMDS. The filters were then stored in a desiccator under gentle vacuum. Finally, the prepared filters were mounted on stubs, sputter coated (Emscope SC500; Ashford, UK) with gold–palladium and viewed at 10 kV using a Scanning Electron Microscope (FEI Quanta FEG 200; Eindhoven, the Netherlands). SEM micrographs were presented on a black background using Photoshop 6.0 (Adobe Systems; San Jose, California, USA).

### 2.4 DNA Extraction, Sequencing and Phylogenetic Analysis

Detailed methods describing the DNA extraction and sequencing of *P*. *insidiosum* sp. nov. are available in Larsson et al. (2022) and for *P. cordatum* strain 1‐B3 in Tillmann, Gottschling, et al. (2023). Sequences are available at GenBank (www.ncbi.nlm.nih.gov) with accession numbers MW024110–MW024113 for the SSU gene region; MW024089–MW02492 for the ITS gene region; and MW024106–MW02409 for the LSU gene region for *P. insidiosum* sp. nov., and PV156052 (ITS); and PV156051 (LSU) for *P. cordatum* strain 1‐B3.

Phylogenetic analyses were conducted in Geneious Prime v2022.0.1 (Biomatters Ltd., Auckland, New Zealand) (https://www.geneious.com). The 18S SSU, ITS, and 28S LSU nuclear rRNA gene regions were analyzed separately, then as concatenated long sequences spanning all three regions (SSU + ITS + LSU).

For the individual gene region phylogenies, publicly available sequences from strains representing the five morphologically similar, small, round, pelagic, *Prorocentrum* species ( *P. cordatum* , *P. pervagatum*, *P. spinulentum*, *P. shikokuense*, *P. thermophilum* ), and other dinoflagellates used as out‐groups, were downloaded from GenBank (www.ncbi.nlm.nih.gov) and aligned with the sequences of *P. insidiosum* sp. nov. from Larsson et al. (2022) using the MUSCLE algorithm (maximum number of iterations 8) (Edgar 2004). Aligned sequences from the SSU, ITS, and LSU gene regions were then truncated to 1439, 557, and 623 bp, respectively, and analyzed separately. Maximum Likelihood (ML) phylogenetic trees were generated for all gene regions using PHYML with 1000 bootstraps (Guindon and Gascuel 2003) using a GTR substitution model and an estimated gamma distribution. Bayesian analysis was performed for all gene regions using MrBayes 3.2.6 (Huelsenbeck and Ronquist 2001) by means of the GTR + G (general‐time reversible with gamma‐shaped among‐site variation) model. Bayesian analyses were carried out in four simultaneous runs with four chains each for 3.1 × 10^6^ generations, sampling every 1000 trees with 1000 trees discarded as burn‐in.

For the concatenated SSU + ITS + LSU phylogeny, sequences from strains of *Prorocentrum* that had at least two of the three gene regions available were downloaded and aligned using the MAFFT algorithm v7.490 (Katoh and Standley 2013) following the methods outlined in Gottschling et al. (2020). Separate alignment matrices were constructed for each gene region (SSU, ITS, LSU) and then concatenated. Accession and strain information of the sequences used for this analysis are available in Table S1. Maximum Likelihood (ML) phylogenetic trees were generated using PHYML v3.3.280621 with 1000 bootstraps (Guindon et al. 2010) using a GTR substitution model and an estimated gamma distribution. Bayesian analysis was performed for all gene regions using MrBayes 3.2.6 (Huelsenbeck and Ronquist 2001) by means of the GTR + G (general‐time reversible with gamma‐shaped among‐site variation) model. Bayesian analyses were carried out in four simultaneous runs with four chains each for 3.1 × 10^6^ generations, sampling every 1000 trees with 1000 trees discarded as burn‐in.

### 2.5 Terminology

Terminology of cell orientation, designation of thecal plates, periflagellar platelet arrangement, and thecal plate surface ornamentation follows Hoppenrath et al. (2013) supplemented by Tillmann et al. (2019).

## 3 Results

### 3.1 Formal Description

**FIGURE 2:**
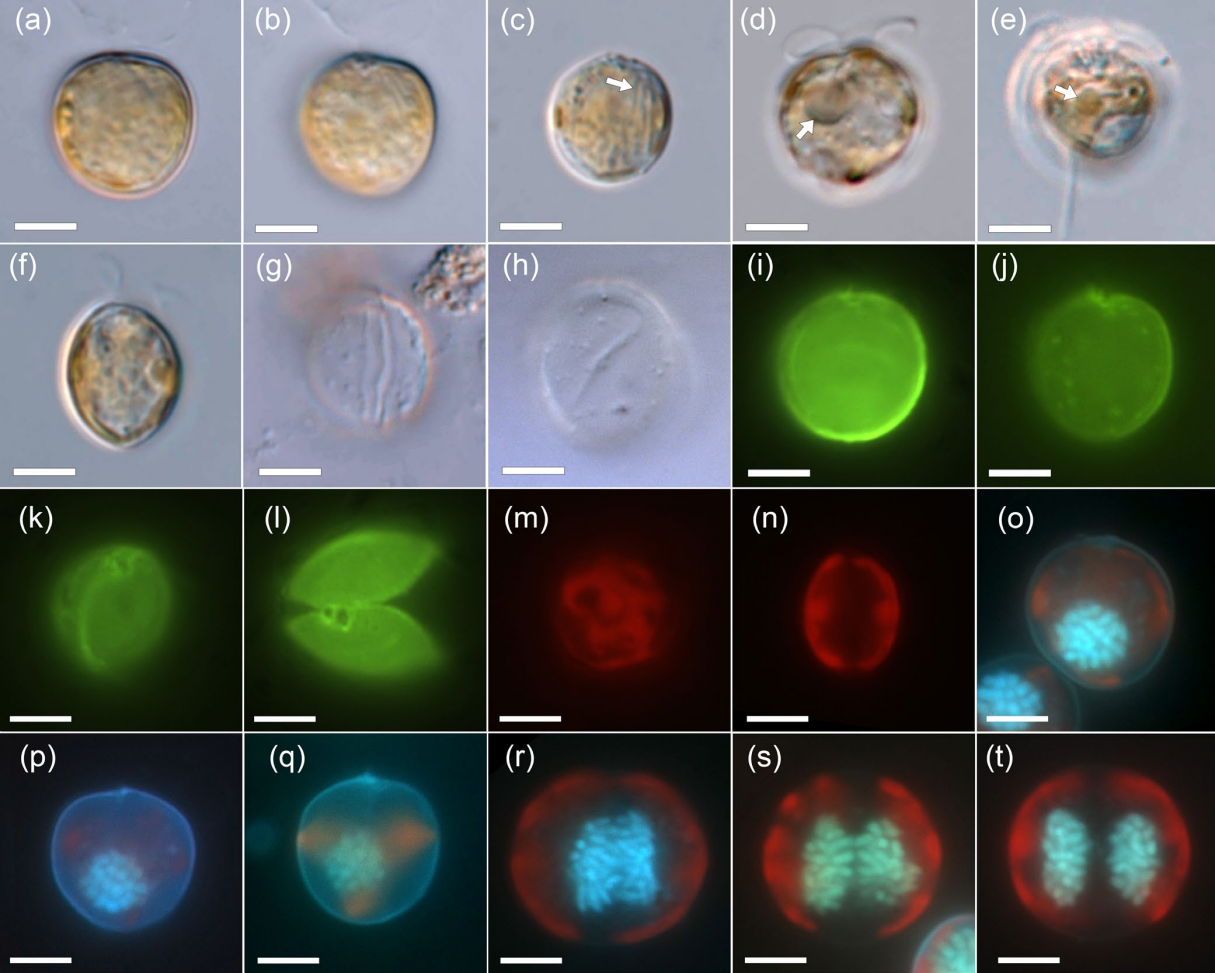
*Prorocentrum insidiosum* sp. nov., strain UTSPH3D3. Light microscope images of living (a–f), or formaldehyde‐preserved cells (g–t). Different cells in lateral view (a–e), or in dorsoventral view (f). Note the rod‐like extrusomes (likely trichocysts) in (c), the hyaline pusule in (d) and the potential pyrenoid in (e) (all denoted with white arrows). (g, h) Empty thecae showing a broad intercalary band between thecal plates (g) and visible pores on the thecal plate surface (h). (i–l) Cells stained with solophenylflavine and viewed with epifluorescence and blue light excitation in lateral (i, j), dorsal‐apical (k), and apical view (l). Note the visible thecal pores in (j) and the accessory and flagellar pore in (l). (m, n) Cells viewed with epifluorescence demonstrating the chloroplast structure (red autofluorescence) throughout the cell in lateral (m) and dorsal/ventral view (n). (o–q) Cells stained simultaneously with calcofluor white and DAPI and viewed with UV excitation to demonstrate the posterior location of the round nucleus (blue). (r–t) DAPI stained cells viewed with UV excitation showing latitudinal elongation of the nucleus during replication prior to cell division. Scale bars = 5 μm.

**FIGURE 3:**
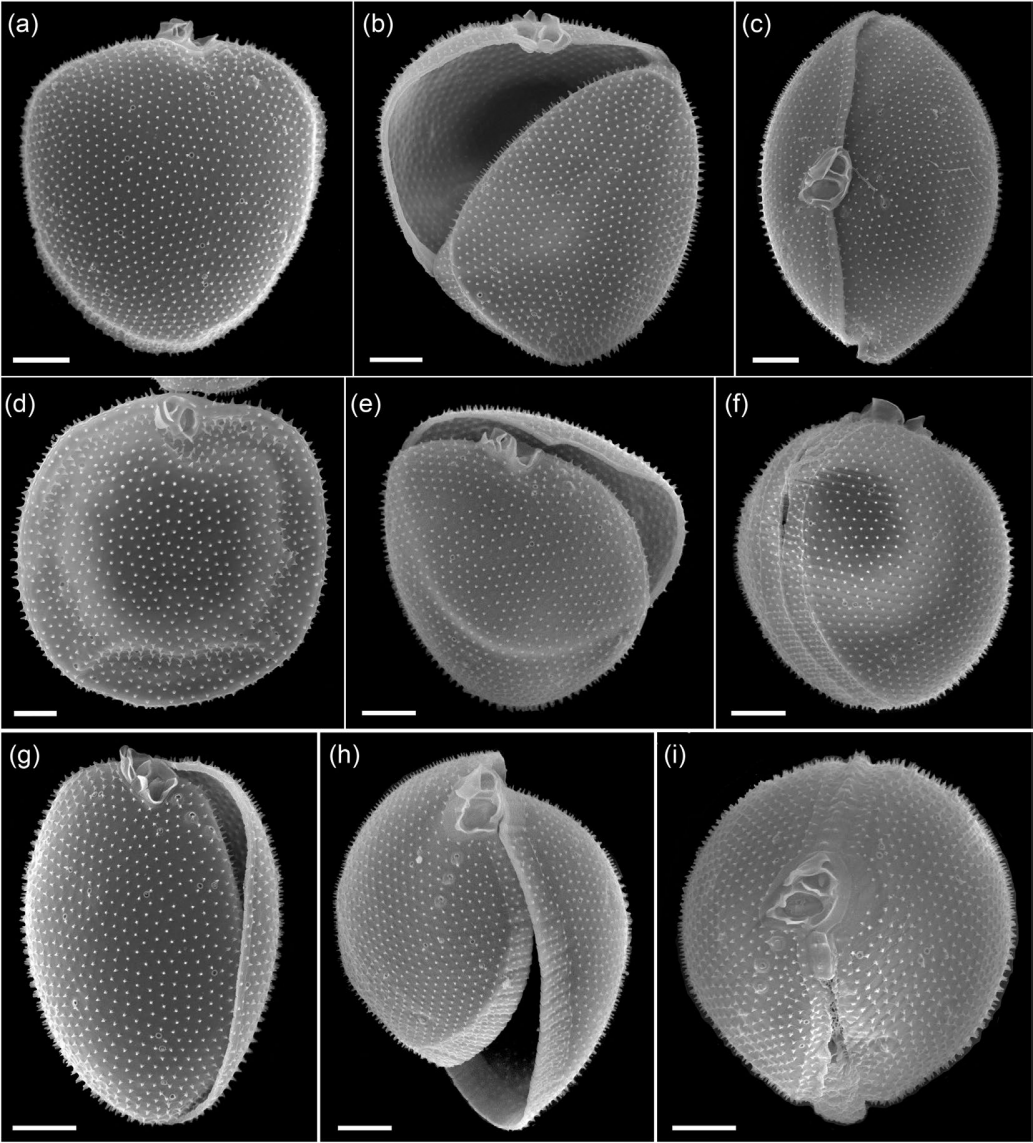
*Prorocentrum insidiosum* sp. nov., strain UTSPH3D3. SEM of different thecae; (a) cell in right lateral view, note the small thecal pores irregularly distributed across the thecal plate surface; (b) cell in left‐thecal view with left and right thecal plates detached; (c) cell in apical view; (d) cell in right‐lateral view; (e) cell in right‐lateral ventral view with left and right thecal plates detached; (f) cell in right‐dorsal view showing the sagittal suture and the wide and transversely striated intercalary band; (g–i) cells in right‐ventral (g), apical ventral (h), and apical view (i) showing the apical projections, small thecal pores and the row of three large pores; (g) sagittal suture without intercalary band often seen in “young” cells. Scale bars = 2 μm.

**FIGURE 4:**
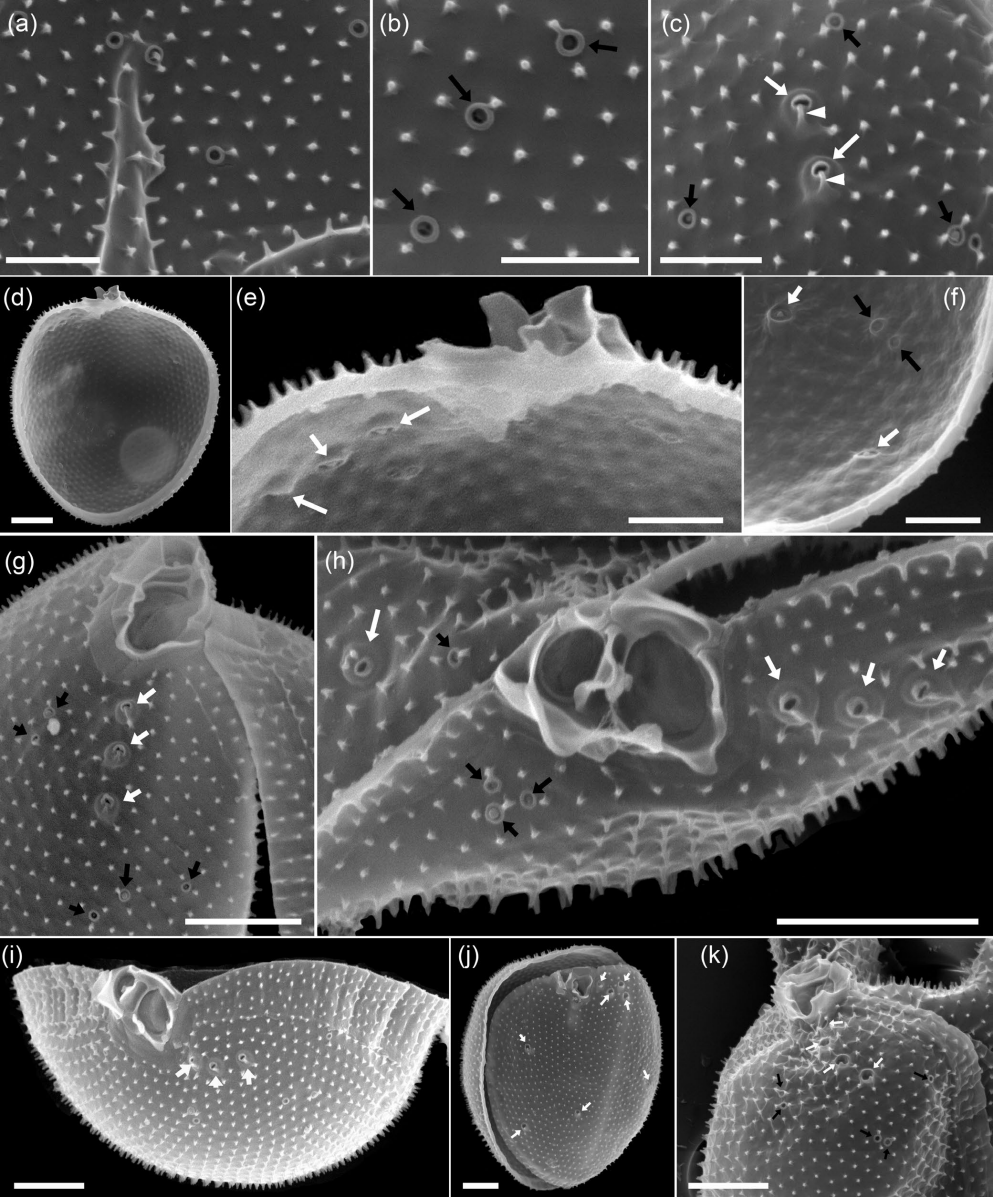
*Prorocentrum insidiosum* sp. nov., strain UTSPH3D3. SEM of different thecae; (a–c) detail of the surface ornamentation and the small thecal pores showing the three‐dimensional structure of the short spine‐like projections (a), the tiny radial extensions emanating from the base of the short spines and the spines positioned close to the rim of the small pores (black arrows) (b), small pores (black arrows) and large pores (white arrows) identifiable by the crateriform rim with additional elevated outer perimetral ring (here partially covered with thecal plate material) and by a closely arciform spine fused with the pore opening (white arrowheads) (c). (d–f) Interior view of the right thecal plate of a cell under multiple magnifications indicating the position of small (black arrows) and large (white arrows) thecal pores. (g, h) Right apical ventral view (g) and apical view (h) of the periflagellar area showing the small (black arrows) and large (white arrows) thecal pores. (i) Apical view of the right thecal plate, note the broad and transversely striated intercalary band and the large pores (white arrows) in apical position. (j) Dorso‐apical view of the right thecal plate detached from the left, note that here all large pores (white arrows) had very large openings and there were four arranged in a cluster in apical ventral position. (k) Apical ventral view of the periflagellar area contrasting the size of the small (black arrows) and large (white arrows) thecal pores, note again the four large pores with large openings arranged in a row in apical ventral position. Scale bars = 1 μm.

**FIGURE 5:**
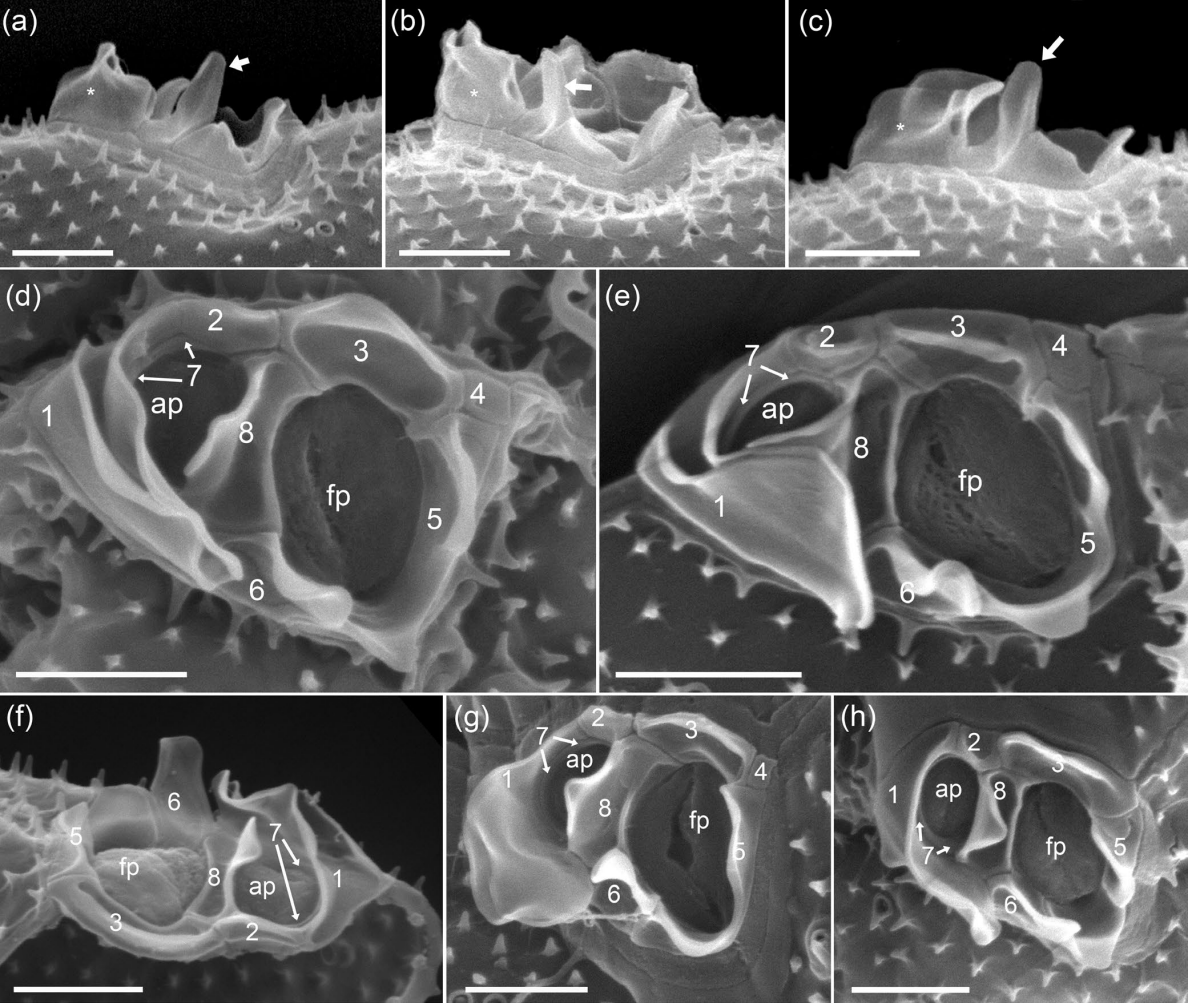
*Prorocentrum insidiosum* sp. nov., strain UTSPH3D3. SEM of the periflagellar area; (a–c) apical projections in right‐lateral view. Dominant in terms of height was a wide wing on the dorsal side (asterisk) and a narrower protrusion in the middle (white arrow). (d–h) Apical view of the periflagellar area showing the detailed shape and arrangement of the large flagellar pore (fp), smaller accessory pore (ap), periflagellar platelets and apical projections including the two conspicuous wings bordering both the right side of platelet 1 and around the accessory pore, and the distinct apical protrusion on platelet 6; note the internal closure of both pores with two lip‐like structures. Numbers denominate the periflagellar platelets. Scale bars = 1 μm.

**FIGURE 6:**
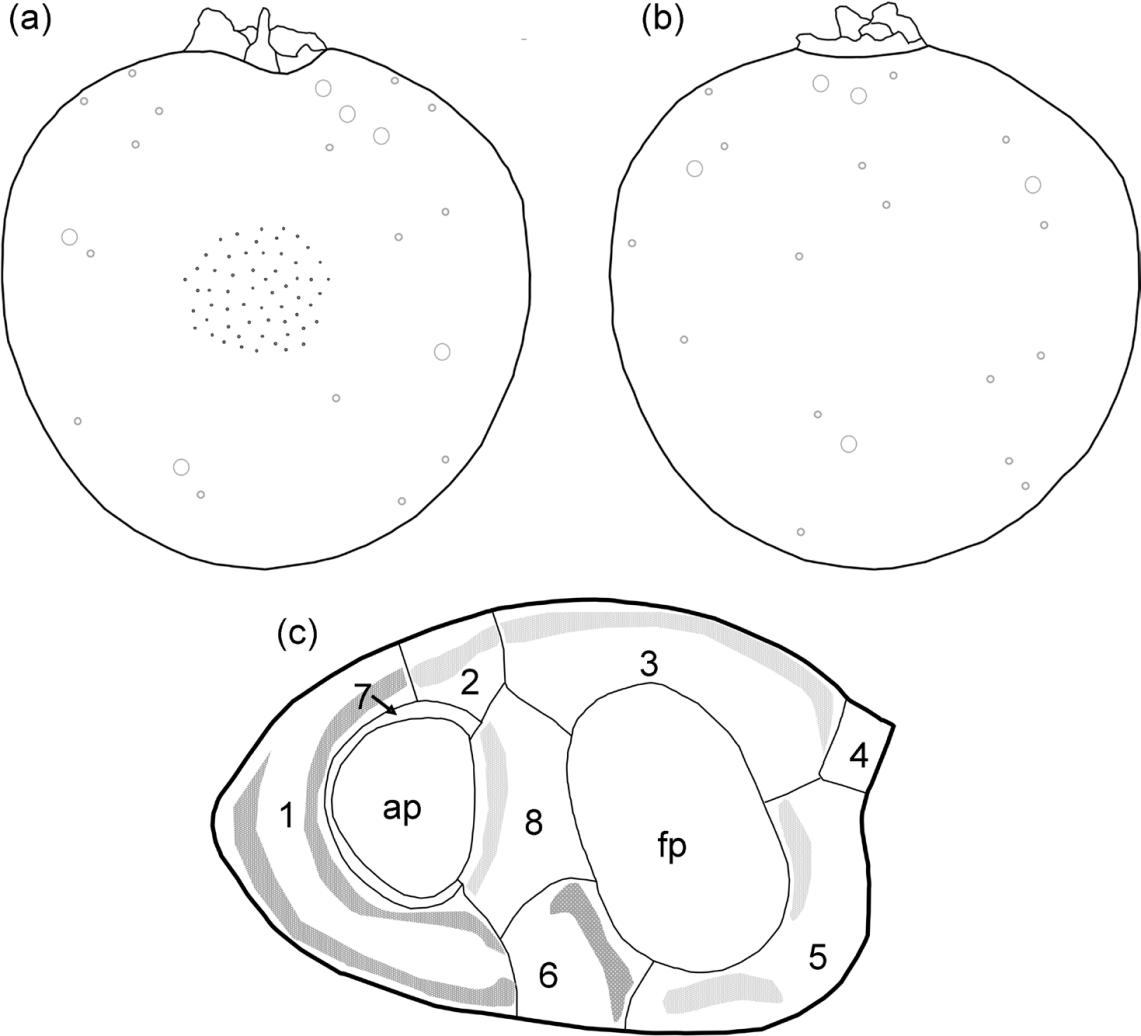
*Prorocentrum insidiosum* sp. nov., schematic drawings of a representative pore pattern of the right (a) and left (b) thecal plates. The density of ornamental spines is indicated in the center of the right thecal plate (a). (c) Schematic drawing of the periflagellar area; numbers indicate denominations of the platelets; ap, accessory pore; fp, flagellar pore; dark gray shows the location of the dominant wings on platelet 1 and protrusion on platelet 6; light gray shows the location of the less prominent wings on platelets 2, 3, 5 and 8.

*Prorocentrum insidiosum* sp. nov. Tillmann, Larsson & Hallegraeff (Figures 1, 2, 3, 4, 5, 6).

*Description*: Small, mixoplanktonic, thecate dinoflagellate with desmokont flagellation; cell outline in lateral view round to slightly ovate. Cell laterally compressed and broadly lens‐shaped in ventral view; posterior end rounded; anterior end round or moderately truncated. Cultured cells 12–16 μm in length and 8–15 μm in depth; wide transversely striated intercalary band on mature cells; round to oval shaped nucleus in a posterior position; two golden‐brown reticulate chloroplasts. Periflagellar area composed of 8 platelets, a small round accessory pore, and a large elongated flagellar pore. Two long conspicuous wings run along the right margin of platelet 1 and around the accessory pore with additional projections on most other platelets except platelet 4. Both thecal plates are densely ornamented with evenly distributed short spine‐like projections. Pores of two size classes are irregularly distributed across the thecal surface; with a row or cluster of two to four large round pores located on the right thecal plate in an apical ventral position.

*Holotype*: SEM‐stub prepared from clonal strain UTSPH3D3 (designated CEDiT2025H202) deposited at the Senckenberg Research Institute and Natural History Museum, Centre of Excellence for Dinophyte Taxonomy, Germany.

*Additional material*: A live culture of strain UTSPH3D3 has been deposited at, and is available from, the Australian National Algal Culture Collection (ANACC) as strain CS‐1390.

*Type locality*: Port Hacking 100 m Australian Integrated Marine Observing System (IMOS) National Reference Station located on the continental shelf of southeast Australia, Pacific Ocean (34.120° S, 151.224° E).

*Habitat*: Marine, planktonic.

*Strain establishment*: Sampled during routine monthly monitoring at the Port Hacking 100 m Australian Integrated Marine Observing System (IMOS) National Reference Station in September 2018. Isolated by M. E. Larsson in October 2018.

*Etymology*: This epithet (*insidiosus* = Latin, adjective meaning insidious, sneaky, crafty) reflects the curious behavior observed in this organism whereby a mucus structure is constructed and used to attract and immobilize prey for heterotrophic consumption.

*Molecular characterization*: The strain UTSPH3D3 is barcoded in GenBank by the sequences of nuclear 18S small subunit (SSU) rRNA gene, nuclear internal transcribed spacer 1 (ITS1)–5.8 S–internal transcribed spacer 2 (ITS2) rRNA gene, D1‐D6 of nuclear 28S large subunit (LSU) rRNA gene under the accession numbers MW024113, MW024091, and MW024109, respectively.

*Phycobank registration*: http://phycobank.org/105393.

### 3.2 Detailed Description

#### 3.2.1 Light Microscopy (LM)

Cells were asymmetrically round to ovate in lateral view (Figure 2a,b) and had a moderate lateral compression (Figure 2f). The posterior end of the cell was round, and the anterior end was round or moderately truncated (Figure 2a,b). Cell size of strain UTSPH3D3 ranged from 11.7 to 16.0 μm in length (mean ± SD, 13.8 ± 1.0 μm, *n* = 30) and 8.0 to 15.2 μm (mean ± SD, 12.0 ± 1.65 μm, *n* = 30) in depth, with a length:depth ratio of 0.96 to 1.48 (mean ± SD, 1.16 ± 0.11). Long rod‐like structures, likely trichocysts, located in apical ventral positions, were visible with LM (Figure 2c). Occasionally, a hyaline pusule was present in the anterior area (Figure 2d). There were no clear signs of the presence of a pyrenoid; although on occasion a small, round, and separate part of the chloroplast could be seen (Figure 2e). Between the plates of empty thecae, the intercalary band was visible (Figure 2g). The thecal plate surface appeared smooth, and the thecal pores were difficult to detect in live or preserved cells with LM but were faintly visible on empty thecae under differential interference contrast (Figure 2g,h) and most easily seen in solophenyflavine‐stained cells under blue light excitation (Figure 2j). Thecal pores were scattered across the thecal plate surface, with a restricted central area free of pores (Figure 2g,h,j). Apical projections were hardly visible in live and preserved cells with LM (Figure 2a–c) but were visible in solephenyflavine‐stained cells under blue light excitation (Figure 2i–l), as were the large flagellar pore and a distinctly smaller accessory pore in apical position (Figure 2l). Two golden‐brown and reticulate chloroplasts were arranged parietally and close to the thecal plate surface (Figure 2f,m,n). The large nucleus was round in outline and located in the posterior region of the cell (Figure 2o–q). During cell division, the nucleus elongated along the latitudinal axis (Figure 2r,s) before chromosomes were eventually separated prior to cell division (Figure 2t).

#### 3.2.2 Scanning Electron Microscopy (SEM)

The two thecal plates were uniformly ornamented with short spine‐like projections (Figures 3 and 4) ranging in length from 0.15 to 0.35 μm (mean ± SD, 0.21 ± 0.04 μm, *n* = 33) (Table 1). At the base of each spine, there were 3–5 tiny radial extensions (Figure 4b). The areal density of the short spine‐like thecal projections was 7.1–8.6 μm^−2^ (mean ± SD, 7.8 ± 0.4 μm^−2^, *n* = 40 counting fields) (Table 1). The intercalary band was variable in width (Figure 3g,h). For broad growth bands, there was a transverse striation consisting of latitudinal rows of the short spine‐like projections that was more visible in mature cells (Figures 3f,h and 4i).

**TABLE 1:** *Prorocentrum insidiosum* sp. nov., strain UTSPH3D3, morphometry of thecal projections and pores. Mean ± standard deviation; minimum to maximum (range); *n* = number of cells measured.

	Spines		Thecal pore size		Number of pores			
	Length (μm)	Density (μm^−2^)	Large (μm)	Small (μm)	Right thecal plate		Left thecal plate	
					Large (μm)	Small (μm)	Large (μm)	Small (μm)
Mean ± SD	0.21 ± 0.04	7.8 ± 0.4	0.41 ± 0.06	0.12 ± 0.01	6.1 ± 1.4	16.6 ± 3.2	5.2 ± 1.4	16.6 ± 2.9
Min. to max.	0.15–0.35	7.1–8.6	0.35–0.5	0.11–0.13	3–9	10–26	3–8	11–21
No. measured	*n* = 33	*n* = 40	*n* = 8	*n* = 20	*n* = 28	*n* = 28	*n* = 19	*n* = 19

There were two types of thecal pores. Small pores were 0.11–0.13 μm (mean ± SD, 0.12 ± 0.01 μm, *n* = 20) in diameter, delimited by a crateriform rim, and were irregularly distributed over the surface of both thecal plates at a density of 10–26 (mean ± SD, 16.6 ± 3.2 plate^−1^, *n* = 28) on the right thecal plate, and 11–21 (mean ± SD, 16.6 ± 2.9 plate^−1^, *n* = 19) on the left (Table 1; Figures 3a,d and 4a–c,g–k). A second type of pore, denominated as large pores, was clearly differentiated from the small pores in interior view where the large pores had a wide diameter and were clearly raised into a crater shape, whereas the small pores were flat in the center (Figure 4d–f). In external view, these large pores, in most cases, had only a small opening delimited by a crateriform rim, but they were still distinguishable from the small pores by their distinct, smooth ring structure surrounding the opening. This gave the impression that the actual opening was overgrown by plate material. In a few cases, the actual opening of the large pores was much larger (Figure 4j,k). Measurements of the large pore diameter, based on the outline of the outer perimetral ring structure regardless of the size of the actual opening, ranged from 0.35 to 0.5 μm (mean ± SD, 0.41 ± 0.06 μm, *n* = 8) (Table 1). Another recognizable feature of the large pores was the presence of an arciform spine that was bent over and fused with the edge of the crateriform rim (Figure 4c). On occasion, the rim of the small pores was connected to a closely positioned spine, but the vertical form of the spine remained unchanged (Figure 4b). Large pores were most obvious as a row, and on occasion a cluster, of two to four (mostly three) in an anterior ventral position on the right thecal plate (Figures 3g–i and 4g–k) but were also irregularly distributed over the surface of both thecal plates. The total density of large pores was three to nine (mean ± SD, 6.1 ± 1.4, *n* = 28) on the right thecal plate and three to eight (mean ± SD, 5.2 ± 1.4, *n* = 19) on the left thecal plate (Table 1). The two types of pores were distinguishable when viewing the interior of a thecal plate.

The periflagellar area (Figures 4g–k and 5) was 2.31–2.79 μm in length (mean ± SD, 2.50 ± 0.13 μm, *n* = 12), 1.53–2.07 μm in width (mean ± SD, 1.75 ± 0.17 μm, *n* = 12) (Table 2) and located between both thecal plates in a broadly V‐shaped indentation at the anterior end of the right thecal plate (Figure 4i). There were eight periflagellar platelets (1, 2, 3, 4, 5, 6, 7, and 8) surrounding a large irregularly ovate flagellar pore (fp) which was 1.06–1.35 μm (mean ± SD, 1.23 ± 0.08 μm, *n* = 12) in length and 0.67–0.96 μm (mean ± SD, 0.81 ± 0.07 μm, *n* = 12) in width, and a smaller accessory pore (ap) 0.64–0.98 μm (mean ± SD, 0.84 ± 0.09 μm, *n* = 12) in length and 0.43–0.68 μm (mean ± SD, 0.58 ± 0.07 μm, *n* = 12) in width (Table 2; Figure 5d–h). Both pores were internally closed by two lip‐like structures (Figure 5d–h). There were multiple apical projections, which were dominated by two conspicuous wings (i.e., projections which were wider than they were long) bordering the area of platelet 1 on both the right side and around the accessory pore. The wing on the right side of platelet 1 was 1.00–1.73 μm (mean 1.36 ± 0.17 μm, *n* = 14) in width and 0.81–1.08 μm (mean 0.92 ± 0.09 μm, *n* = 16) in height (Table 2; Figure 5d,e). There was another distinct apical protrusion (i.e., a projection that was higher than it was wide) located on platelet 6 (Figure 5d–h) that was approximately the same height as, but far narrower than, the wings. Other platelets also had wings, so the accessory and flagellar pores appeared encircled by such projections (Figure 5d–h). Exceptions were platelet 8 where there was a distinct wing‐like projection towards only the accessory pore, and the small and rectangular platelet 4 which was consistently free of projections (Figure 5d–h).

**TABLE 2:** *Prorocentrum insidiosum* sp. nov., strain UTSPH3D3, morphometry of periflagellar area. Mean ± standard deviation, minimum to maximum (range), *n* = number measured. Measurements in μm.

	Periflagellar area		Accessory pore		Flagellar pore		Right wing on platelet 1	
	Length	Width	Length	Width	Length	Width	Width	Height
Mean ± SD	2.5 ± 0.13	1.75 ± 0.17	0.84 ± 0.09	0.58 ± 0.07	1.23 ± 0.08	0.81 ± 0.07	1.36 ± 0.17	0.92 ± 0.09
Min. and max.	2.31–2.79	1.53–2.07	0.64–0.98	0.43–0.68	1.06–1.35	0.67–0.96	1.00–1.73	0.81–1.08
No. measured	*n* = 12	*n* = 12	*n* = 12	*n* = 12	*n* = 12	*n* = 12	*n* = 14	*n* = 16

Schematic drawings of the new species *P. insidiosum* sp. nov., including a representative pore pattern and a schematic drawing of the periflagellar region, are presented in Figure 6.

For a thorough, morphological comparison of *P. insidiosum* sp. nov. with the closely related *P. thermophilum* , two strains of *P. thermophilum* from the CCMP culture collection (CCMP1787 and CCMP1260) were analyzed with light and electron microscopy. Micrographs and the accompanying morphological descriptions are compiled in the Supporting Information (Figures S1–S5). A clear description of the periflagellar platelets was not provided in the protologue of *P. thermophilum* and is thus presented here. Briefly, the periflagellar area of both strains of *P. thermophilum* was composed of eight platelets surrounding the accessory and flagellar pores (Figures S3–S5). On platelet 1, there were several flat wings which were variably arranged, mostly parallel, delineating the accessory pore (Figures S4 and S5h–j). Excluding flat rims surrounding both pores, there were no prominent apical projections.

**FIGURE 7:**
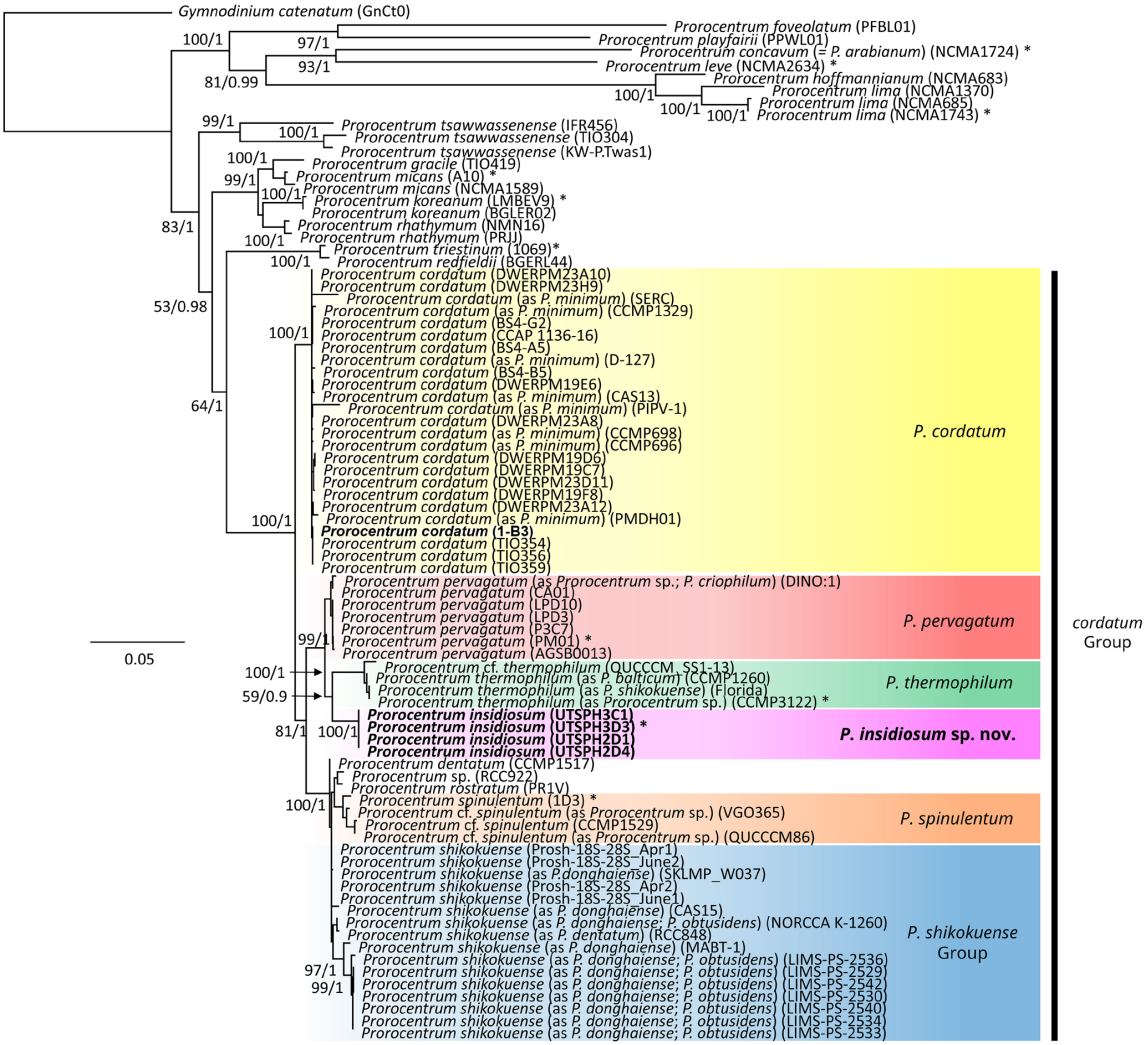
Maximum Likelihood (ML) phylogenetic tree showing alignment of 83 nuclear SSU + ITS + LSU rRNA gene sequences (623 bp) demonstrating the sequences from the four *Prorocentrum insidiosum* sp. nov. strains were located within the *cordatum* group and were distinct from all genetically represented species within the genus *Prorocentrum*. The genus, species and strain code are provided for each sequence, and colored boxes represent species delimitations. Sequences from this study are shown in bold type. Species classifications represent current assignment, with the original species classification as it appears in GenBank, also provided. Species type, or reference material, are indicated by an asterisk. Values at nodes represent ML bootstrap/Bayesian support. Values under 50 and 0.90, respectively, are not shown. The scale bar 0.05 represents substitutions per site.

Morphological observations of the phylogenetically confirmed strain of *P. cordatum* (1‐B3) (Figure 7) revealed the presence of both large and small pores for this species, a previously unconfirmed feature. In addition, the *P. cordatum* strain 1‐B3 had a row of two large pores in apical position on the ventral side of the right thecal plate (Figure S6g–i). A more detailed description of the morphology of this strain is compiled in the Supporting Information and presented in Figure S6.

#### 3.2.3 Molecular Phylogeny

The phylogenetic position of *P. insidiosum* sp. nov. was inferred from three nuclear rRNA markers including 18S SSU, ITS, and partial 28S LSU (D1–D6) regions. Each gene region was analyzed individually (Figures S7–S9), and as concatenated long sequences spanning all three regions (SSU + ITS + LSU) when sequences from at least two regions were available for a given strain (Figure 7). The topologies of the Bayesian‐inference and Maximum Likelihood phylogenies were congruent, and most clades received high bootstrap and posterior probability support. Phylogenetic analysis of the SSU, ITS, LSU, and SSU + ITS + LSU gene regions showed *P. insidiosum* sp. nov. formed a monophyletic lineage distinct from all genetically represented species within the genus *Prorocentrum* (Figure 7, Figures S7–S9).

Analysis of SSU sequences (1439 bp) from 42 strains representing the six morphologically similar, small, round, pelagic, *Prorocentrum* species (denoted the *cordatum* group) showed separation into two distinct clades (Figure S7). The first clade had two highly supported lineages consisting of the *P. insidiosum* sp. nov. sequences (99/1) and another with sequences representing the species *P. thermophilum* (89/0.99). The second clade was comprised of four lineages. One lineage contained sequences that represent the species *P. cordatum* (86/0.91), while another separated into sub‐lineages, each with sequences representing the species *P. pervagatum*, *P. spinulentum*, and the *P. shikokuense* group (72/1; 84/1; 80/0.93, respectively).

The topology and cladal separation of the ITS phylogeny was similar to the SSU, though some lineages were less well resolved (Figure S8). The 89 ITS sequences (557 bp) representing individual strains from the six morphologically similar *Prorocentrum* species within the *cordatum* group showed separation into two poorly supported clades (48/0.89), with sequences from *P. insidiosum* sp. nov. located in the second. The first clade comprised 32 sequences of *P. cordatum* , while the second consisted of four lineages with sequences representing the remaining five species. One lineage consisted of sequences from the *P. shikokuense* group (86/1) with sequences from *P. spinulentum* embedded within (98/0.84) and another with sequences representing *P. pervagatum* (87/1). A third lineage contained two sub‐lineages with sequences representing the species *P. insidiosum* sp. nov. (99/1) and *P. thermophilum* (80/0.99) (Figure S8).

The topology of the LSU gene phylogeny was analogous to that of the SSU and ITS phylogenies, showing each of the six morphologically similar *Prorocentrum* species within the *cordatum* group, including *P. insidiosum* sp. nov., separated into individual lineages with high support (Figure S9). The LSU phylogeny, including sequences (623 bp) from 60 strains, showed separation into three distinct clades with sequences from *P. insidiosum* sp. nov. located in the third. The first clade separated into a monophyletic clade of sequences from the species *P. cordatum* . The second included two highly supported (88/0.98) lineages of sequences representing the species *P. spinulentum* (66/0.97) and the *P. shikokuense* group (89/1). The *P. shikokuense* group lineage also showed sub‐lineages within. The third clade was comprised of a highly supported lineage of sequences representing the species *P. pervagatum* (96/1) and another that split into two sub‐lineages representing *P. insidiosum* sp. nov. (100/1) and *P. thermophilum* (70/0.96) (Figure S9).

The SSU + ITS + LSU phylogeny incorporated sequences from 83 strains and included both the six morphologically similar *Prorocentrum* species within the *cordatum* group, in addition to other species from the genus more broadly (Figure 7). The species from the *cordatum* group separated into distinct clades with full support (100/1). Within this clade, the sequences from strains of *P. insidiosum* sp. nov. formed a monophyletic group with full support (100/1) (Figure 7).

## 4 Discussion

The description of *P. insidiosum* sp. nov. provides a scientific name for the small, pelagic, mucosphere‐producing, mixoplanktonic dinoflagellate from Larsson et al. (2022). This species is phylogenetically distinct from all genetically represented species within the genus *Prorocentrum* and morphologically differentiated by a specific combination of characteristics. Namely, cell size and shape; the number and shape of apical projections; the number of periflagellar platelets; the length of thecal plate ornamental projections with the presence of radial extensions; and the form and position of both small and large thecal pores.

The challenge with describing a new small, pelagic, roundish *Prorocentrum* species is differentiating it from the ~20 similar taxa, of which many are historical determinations that have incomplete descriptions and lack ultrastructural details of the periflagellar platelet pattern and physical type material. However, six of the more recent species determinations ( *P. cordatum* , *P. nux* , *P. pervagatum*, *P. shikokuense*, *P. spinulentum*, *P. thermophilum* ) include phylogenetic analysis and/or detailed morphological descriptions, which provide a solid foundation for a thorough diagnostic and comparative discussion.

Molecular phylogenetic analysis showed the species most closely related to *P. insidiosum* sp. nov. is the recently described *P. thermophilum* , a species originating from the warm waters of the Atlantic, Indian, and Pacific Oceans whose strains (e.g., CCMP1260 and CCMP1787) were previously referred to as *Prorocentrum balticum* (Lohmann) Loeblich III (Gómez et al. 2023). A clear description of the periflagellar platelets was not included in the protologue of *P. thermophilum* , so additional SEM micrographs of the apical region of strains CCMP1260 and CCMP1787 were collected as part of this study for comparison (Figures S1–S5). While *P. thermophilum* and *P. insidiosum* sp. nov. share the same cell size, *P. thermophilum* is far less laterally compressed, and the thecal plate projections are shorter, more knob‐like, and less dense than the spines of *P. insidiosum* sp. nov. (Table 3). The periflagellar projections also differ in *P. thermophilum* in that they are much smaller, less prominent, and more complex, forming several flat wings on platelet 1 and surrounding the accessory pore (Figure S4).

**TABLE 3:** Morphological comparison of small, pelagic *Prorocentrum* species that are closely related to *P. insidiosum* sp. nov. based on molecular phylogenetic analysis.

Feature	*P. insidiosum*	*P. thermophilum*	*P. pervagatum*	*P. spinulentum*	*P. shikokuense*	*P. cordatum*
Cell shape in lateral view	Round to ovate	Almost round	Asymmetrically oval to round	Irregularly oval to round	Similar to a spatula without a handle^a^	Ovate, Rounded ovate^b^
Degree of lateral compression^c^	Moderate	Minor, if at all	Moderate	Moderate	Strong	Strong
Cell length (mean and min to max range) (μm)	13.8 (11.8–16.0)	13.9 (11.8–16.6)	10.6–13.9^d^ (8.3–16.0)	10.9 (9.0–12.8)	(15.3–19.9)	(10–18)^e^
Cell depth^f^ (mean and min to max range) (μm)	12.0 (8.0–15.2)	12.3 (9.8–15.2)	10.2–14.6^d^ (8.0–15.9)	10.3 (8.5–11.9)	(8.1–9.4)	(10–18)^e^
Shape and position of the nucleus	Round/oval, posterior	Round/oval, posterior	Round/oval, posterior	Oval, median/sub‐median	Round, posterior	Round/oval, posterior
Thecal plate surface ornamentation	Knob‐spines	Knobs	Knobs	Spines	Knobs‐spines	Spines
Thecal plate surface projection density (mean and min to max range)	7.8 (7.1–8.6)	5.9 (5–7)	7.0–10.5^d^ (6.41–12.7)	4.6 (4.2–5.0)	10.6 ± 1.1	(3.9–4.5)^g^
Thecal plate surface projection length (mean and min to max range) (μm)	0.21 (0.15–0.35)	n.d.	0.10–0.17^d^ (0.07–0.25)	0.36 (0.30–0.43)	Unclear	(0.37–0.40)^g^
Radial extensions from thecal plate surface projections	Minor	Strong	None	Medium	Minor‐medium	None^h^
Small pores present	Yes	Yes	Yes	Yes	Yes	Yes
Distinct rim around small pores	Yes	Yes, mostly	No, sometimes faintly	Yes	Yes	No, sometimes faintly
Large pores present	Yes	Yes	Yes	Yes	Yes^i^	Yes^j^
Large pores overgrown	Mostly	At times	At times	At times	Yes^k^	At times^j^
Position of pores	Irregularly scattered over plates	Scattered on plate margins	Scattered on plate margins	Irregularly scattered over plates	Irregularly scattered over plates	Not reported
Apical row of large pores	2–4	3	3–4	3	Not reported	2–3
Number of pores per plate^l^	Approx. 22	Approx. 15^m^	Approx. 25	Approx. 30	n.d.	12–30^n^
No. of periflagellar platelets	8	8	8	8	9 (5a, b)	8^o^
Prominent apical projections	Long and wide double wing on platelet 1	Two or more parallel flat wings on platelet 1	Flat double wing on platelet 1; long spine on 5	Double wing on platelet 1, the outer wing being long and tall	Prominent protrusion on platelet 1 (ear‐shaped collar)	Tall (double) wing on platelet 1; spines on 5 and 8^p^
References	This study	This study and Gómez et al. (2023)	Tillmann, Wietkamp, et al. (2023)	Tillmann, Gottschling, et al. (2023)	Lu and Goebel (2001), Roselli et al. (2019) and Shin et al. (2019)	This study, Pertola et al. (2003), Monti et al. (2010) and Pei et al. (2022)

^a^ This lists the original description of shape by Hada (1975). Other shape descriptions from the literature include “elongated” (Shin et al. 2019) or “sunflower seed‐shaped” (Gómez et al. 2021).

^b^ Also triangular and heart‐shaped, see Monti et al. (2010).

^c^ The degree of lateral compression can vary with the age of the cells, so the categories provided here represent estimates from the appearance of most cells within a culture.

^d^ Represents the range of means reported for different strains, see Tillmann, Wietkamp, et al. (2023), their tables 2,3 and 4.

^e^ Range of six strains, see Monti et al. (2010), their table 2.

^f^ Often referred to as width in the literature.

^g^ Based on three strains from Pertola et al. (2003).

^h^ However, see Pertola et al. (2003), who reported that in some Baltic Sea specimens, the spines are connected to each other with small ridges.

^i^ Both Lu and Goebel (2001) as well as Roselli et al. (2019) report two types of pores referred to as trichocyst pores and valve pores.

^j^ Present study and Tillmann, Mitra, et al. (2023).

^k^ Diameter of “trichocyst pores” are reported as 0.25 μm (Lu et al. 2005; Roselli et al. 2019), whereas in the species description of *P. donghaiense* a diameter of 0.43 μm is reported (Lu and Goebel 2001).

^l^ The number of pores is variable and differs between left and right thecal plates. The values shown are approximate.

^m^ Based on measurements of strains CCMP1260 and CCMP1787 from SEMs completed as part of this study (Figures S1–S5).

^n^ Based on Pertola et al. (2003), who did not report pores per plate but stated that there were 25–59 pores per cell (*n* = 3).

^o^ This study and Pei et al. (2022).

^p^ This study and Pei et al. (2022). Also see Pertola et al. (2003) for a thorough discussion on the different terms used in the literature describing apical projections of *P. cordatum* .

*Prorocentrum pervagatum* (=*P. criophilum*) is widely distributed throughout polar and temperate regions (Tillmann, Wietkamp, et al. 2023) and has a similar cell size, shape, and degree of lateral compression to *P. insidiosum* sp. nov. Features that are distinct from *P. insidiosum* sp. nov. are the location of the thecal pores towards the plate margins; the lack of radial extensions from the thecal plate surface projections; and—most significantly—the presence of an apical projection in the form of a long spine on platelet 6 (Table 3).

*Prorocentrum spinulentum* (Tillmann, Gottschling, et al. 2023) is the most recently described of the small, pelagic *Prorocentrum* species. It has many features that differentiate it from *P. insidiosum* sp. nov. The cells are smaller; the nucleus is oval shaped and in a median to submedian position on the cell's ventral side; the thecal plate surface is covered by long spines (a characteristic that inspired the name of this species). Furthermore, in *P. spinulentum* the outer wing on platelet 1 is very prominent and much higher than the inner wing, whereas in *P. insidiosum* sp. nov., these two bands are of approximately the same height (Figure 5a–c).

There is considerable taxonomic debate surrounding the species *Prorocentrum shikokuense* and its potential synonymy with *P. dentatum* F.Stein, *P. obtusidens* J.Schiller, and *P. donghaiense* D.Lu (Takano and Matsuoka 2011; Shin et al. 2019; Gómez et al. 2021). We agree with the proposition that *P. donghaiense* is synonymous with *P. shikokuense* (Takano and Matsuoka 2011), and that *P. dentatum* and *P. obtusidens* , as described by Schiller, are separate but yet molecularly undefined species based on the morphological comparison described in Gómez et al. (2021). Notably, the currently publicly available sequences for these species do not form clearly defined phylogenetic lineages (Figure 7; Figures S7–S9) and are therefore considered a species group (referred to as the *P. shikokuense* species group) (Gómez et al. 2021; Tillmann, Wietkamp, et al. 2023). Nevertheless, the shape of the cells comprising this group differs from those of *P. insidiosum* sp. nov. in that they are distinctly elongate (Table 3).

*Prorocentrum cordatum* has a widespread distribution and is by far the most intensely studied small, pelagic *Prorocentrum* species (Heil et al. 2005; Glibert et al. 2008; Kalvelage and Rabus 2024). The taxonomy of this species is complex, though most taxonomists agree that *P. cordatum* is the senior synonym of *P. minimum* (Pavillard) J. Schiller (Velikova and Larsen 1999). There is no detailed morphological or phylogenetic characterization available for *P. cordatum* or *P. minimum* from type material from the Caspian Sea or Golf de Lyon (France), respectively. However, information from other strains denominated as either *P. cordatum* or *P. minimum* is available for comparison (strain 1‐B3: this study, Figure S6; Monti et al. 2010; Pei et al. 2022; Pertola et al. 2003). These strains are phylogenetically distinct; the cells are larger and more laterally compressed; the thecal plate surface is less densely ornamented; and the thecal plate spines are longer than those of *P. insidiosum* sp. nov. (Table 3).

*Prorocentrum nux* is a species for which a detailed morphological description is provided in the protologue, but phylogenetic analysis has not been completed (Puigserver and Zingone 2002). This species differs from *P. insidiosum* sp. nov. in that the cells are considerably smaller; the thecal plate surface is smooth (lacks ornamentation); and there are very few (1–3) pores per plate (Puigserver and Zingone 2002). The periflagellar area was described as consisting of seven platelets in total, further differentiating it from *P. insidiosum* sp. nov., which has eight, though it is difficult to determine if the often very small platelet 7 was overlooked in the original analysis (Puigserver and Zingone 2002).

There are quite a number of other small, pelagic *Prorocentrum* species whose original descriptions were based solely on light microscope observations and whose type material consists only of simple line drawings. This makes taxonomic comparison challenging; however, both Tillmann, Wietkamp, et al. (2023) and Gómez et al. (2023) provide compilations of the published drawings along with detailed descriptions, analysis, and comparisons. Some of these species have been synonymized with others in the past (Dodge 1975). However, considering the increasingly apparent molecular phylogenetic diversity of morphologically similar *Prorocentrum* species, many of these synonymizations can be questioned. Therefore, we have included a comparison of these species in our delimitation of *P. insidiosum* sp. nov.

One of the most frequently identified of these species is *P. balticum* . The original description from Kiel (German Baltic Sea) provides only the size (9–12 μm) and shape (slightly ovoid in lateral view and almost round in ventral view) and notes the visible sagittal suture (Lohmann 1908). Apical projections, thecal plate surface ornamentation and thecal pores are not depicted or described, indicating that such features are either not prominent or are not discernible when viewing the cells using light microscopy. There is also variation in subsequent descriptions of strains denominated as *P. balticum* from different locations, further adding to the confusion (Wulff 1916; Adachi 1972; Steidinger and Tangen 1997). A complete phylogenetic and morphological characterization using material from the type locality is required to resolve this discordance, but repeated sampling in the Kiel Bight in recent years has not succeeded in obtaining cells conforming with the Lohmann (1908) protologue (Tillmann, Wietkamp, et al. 2023). We therefore adhere to the original description of *P. balticum* by Lohmann (1908) and consider the small size (9–12 μm), the round shape, and the lack of apical projections as sufficient to differentiate *P. balticum* from *P. insidiosum* sp. nov. Other taxa including *Exuviaella aequatorialis* Hasle and *P. pomoideum* Bursa were considered synonymous with *P. balticum* (Dodge 1975), though this synonymization is now questioned (Tillmann, Wietkamp, et al. 2023). Nonetheless, *E. aequatorialis* is much larger than *P. insidiosum* sp. nov. (19 μm in length according to Hasle) and is markedly compressed (Hasle 1960). Similarly, *P. pomoideum* is described as having a “flattened” cell compression and has distinct structures, either pores or spines, on the thecal plate surface (figure 7d in Tillmann, Wietkamp, et al. 2023), which differ from the dense presentation of small spines observed in *P. insidiosum* sp. nov.

There are also several taxa synonymized with *P. cordatum* , including *Exuviaella pacifica* Kuz'mina, *P. cordiforme* Bursa, *P. marielebouriae* (Parke & Ballentine) A.R. Loeblich III, and *P. triangulatum* G.W. Martin (for detailed information on the size and shape of these taxa see Gómez et al. 2023, Table 2 and Figure S1). However, analysis of material from type localities is required to confirm the status of these taxa. Nevertheless, these taxa are either significantly larger ( *E. pacifica* , *P. triangulatum* ) or smaller (*P. cordiforme*) than *P. insidiosum* sp. nov., have differently shaped cells (*P. cordiforme* is heart‐shaped), or possess distinct features such as a marked notch in the periflagellar area ( *E. pacifica* ). The cell size of *P. marielebouriae* (10–22 μm in length) does overlap with the size of *P. insidiosum* sp. nov., but the species differs in that newly divided cells are strongly laterally compressed and have distinct (stainable with dilute iodine) pyrenoids (Parke and Ballantine 1957).

Contrary to the ovate cell shape of *P. insidiosum* sp. nov., *P. ovum* J. Schiller has elongated ovate shaped cells with a square anterior end (figure 7i in Tillmann, Wietkamp, et al. 2023), and cells of *P. cornutum* J. Schiller have a characteristic horn‐like pointed tip (figure 7k in Tillmann, Wietkamp, et al. 2023). *Prorocentrum nanum* J. Schiller cells are smaller and strongly compressed (figure 7g in Tillmann, Wietkamp, et al. 2023). This species is also considered by some to be synonymous with *P. pusillum* (J. Schiller) J.D. Dodge & Bibby (Dodge 1975; Dodge and Bibby 1973) though this has been questioned (Tillmann, Wietkamp, et al. 2023; Puigserver and Zingone 2002). Nonetheless, the small size and strong compression of *P. pusillum* and *P. nanum* distinguish these species from *P. insidiosum* sp. nov. By contrast, *Prorocentrum antarcticum* (Hada) Balech (figure 7m,n in Tillmann, Wietkamp, et al. 2023) is larger and lacks apical projections making it quite different from *P. insidiosum* sp. nov. Both *P. sphaeroideum* J. Schiller and *P. rotundatum* J. Schiller have a single and solid spine and thus differ from *P. insidiosum* sp. nov., and *P. rotundatum* is larger (16–21 μm) and rounder than *P. insidiosum* sp. nov. (figure 7j in Tillmann, Wietkamp, et al. 2023). Finally, *P. ponticum* Krakhmalny & Terenko has a warty and not spiny plate surface ornamentation (Krakhmalny and Terenko 2004) and a single line of thecal pores located at the periphery of each thecal plate (figure 7p in Tillmann, Wietkamp, et al. 2023) differentiating it from *P. insidiosum* sp. nov.

With the addition of this detailed morphological description of *P. insidiosum* sp. nov., it is now possible to further discuss both the similarities and differences among *Prorocentrum* species within the phylogenetically defined *cordatum* group consisting of *P. cordatum* , *P. pervagatum*, *P. shikokuense*, *P. spinulentum*, and *P. thermophilum* (Figure 7, Figures S7–S9). Regarding cell shape, most species within the *cordatum* group are roundish to oval in lateral view, though *P. shikokuense* deviates with its notable elongation, making the strong phylogenetic relatedness of the small, round *P. spinulentum* with the elongated *P. shikokuense* particularly striking (Tillmann, Gottschling, et al. 2023). Phylogenetic sequences are not currently available from reliably identified strains of the similarly elongated species *P. obtusidens* and *P. dentatum* , and it will be interesting to see when they do become available, which genetic lineage these species belong to. Notably, all species within the *cordatum* group have similar ornamentation of the thecal plate surface with knob or spine‐like structures, which, however, do show species‐specific differences in both length and density. This spiny thecal surface composition is clearly distinct from the smooth plate surface of species from the *P. triestinum* group (Tillmann et al. 2022) and the foveate surface structure found in species from the *P. micans* group (Tillmann et al. 2019).

In addition, species within the *cordatum* group exhibit significant similarities in the structure of the periflagellar region (when also considering the analysis presented in this study for *P. thermophilum* and *P. cordatum* in the Supporting Information; Figures S2–S6). All species display very similar double wing projections on platelet 1, which for *P. cordatum* has been termed a “double‐layered, curved collar” (Pertola et al. 2003). Despite the similarity, the specific configuration of the ornamentation on this platelet can aid in distinguishing between species. The species also possess eight platelets, with the exception of *P. shikokuense*. A division of platelet 5 has been described for this species though this division is not clearly visible in the available SEM micrographs (figure 5 in Shin et al. 2019) and further investigations are needed to determine whether this truly represents a fundamental difference in the number of periflagellar plates within this group. Understanding this is important for assessing whether the periflagellar plate pattern can serve as a general morphological characteristic for defining larger phylogenetic lineages within *Prorocentrum*.

Another common feature of the species within the *cordatum* group is the type of thecal pores. All species exhibit two distinct types of pores, small and large, with a particularly notable feature being a group or row of large pores located in an apical position on the ventral side of the right thecal plate. For *P. cordatum* most studies haven't specifically reported two pore sizes. This is likely because pores are difficult to detect on the spiny thecal plate surface. Nevertheless, as documented in Tillmann, Mitra, et al. (2023) and the present study (Figure S6) and is also visible (but not described) in micrographs presented by Hajdu et al. (2005), large pores are present in *P. cordatum* and form a short row of 2–3 pores in apical position. For *P. pervagatum*, *P. thermophilum* , *P. spinulentum*, and *P. cordatum* , the large pores in most cases are clearly distinguishable from the small pores in terms of the shape and diameter, particularly in interior view. However, for *P. insidiosum* sp. nov., the large pores, which are evident and distinguishable from the small pores in interior view, often only have a small diameter genuine opening in exterior view. The reasons for this remain unclear. It is possible that varied pore opening sizes reflect different temporal states after the expulsion of ejectosomes (as discussed, e.g., for *P. micans* by Tillmann et al. 2019) and thus may be affected by fixation or SEM preparation techniques. In this context, it is worth examining the pores of *P. shikokuense* more closely. In Shin et al. (2019), small pores with a diameter of 0.15 μm are reported and it is explicitly stated that “there were no different types of thecal pore present.” However, in the thecal plate SEM interior views (figure 4 in Shin et al. 2019), two distinctly different pore types with different forms and diameters are visible. Other studies on *P. shikokuense* clearly refer to two different pore types, where the large pores are described as “trichocyst pores” and the small pores as “valve pores” (Lu and Goebel 2001; Roselli et al. 2019). In these studies, SEM interior views of the thecal plates also clearly show two distinct types of pores. The diameter of the large pores is reported as 0.25 μm (Lu et al. 2005; Roselli et al. 2019), whereas Lu and Goebel (2001), in the original species description of *P. donghaiense*, report a diameter of 0.43 μm. SEM images of the exterior surfaces of *P. shikokuense* plates (Lu et al. 2005, Roselli et al. 2019) consistently show large pores with a structure featuring a distinct but closed, smooth circular area and a tube‐like elevated structure with only a small genuine opening, which to some extent resembles the features observed for large pores in *P. insidiosum* sp. nov. Thus, further studies are needed to investigate the detailed structure and function of the different pore types in species of the *cordatum* group.

Finally, it is noteworthy that mucus trap production and mixoplanktonic feeding have now been observed in three species of the *cordatum* group, that is, *P. insidiosum* sp. nov., *P. cordatum* , and *P. pervagatum* (Larsson et al. 2022; Tillmann, Mitra, et al. 2023). Further studies will reveal whether other species in the group also exhibit these fascinating behaviors.

In conclusion, this more detailed examination and comparison of the phylogenetic and morphological characteristics of the Australian strains of *Prorocentrum* provisionally designated as *P*. cf. *balticum* by Larsson et al. (2022) has revealed they are indeed a novel species, *P. insidiosum* sp. nov. This description of yet another new species is helping to clarify the taxonomic confusion within the *Prorocentrum* genus; however, continued effort using the epitypification approach based on cell material from the corresponding type localities is required to resolve enduring issues from ambiguous historical species descriptions and is critical for understanding the biogeography and ecology of this genus.

## Author Contributions

**Michaela E. Larsson:** conceptualization (equal); data curation (equal); formal analysis (equal); funding acquisition (equal); investigation (equal); methodology (supporting); resources (supporting); visualization (supporting); writing – original draft preparation (equal). **Gustaaf Hallegraeff:** conceptualization (equal); writing – review and editing (equal). **Martina A. Doblin:** conceptualization (equal); supervision (lead); funding acquisition (equal); writing – review and editing (equal). **Urban Tillmann:** conceptualization (equal); data curation (equal); formal analysis (equal); funding acquisition (equal); investigation (equal); methodology (lead); resources (lead); validation (lead); visualization (lead); writing – original draft preparation (equal).

## Supporting information


**Figure S1.** *Prorocentrum thermophilum* strain CCMP 1787. Light microscope images of formaldehyde‐preserved cells (a–o). Different cells in lateral view (a–d, g, m, n), in apical view (e, k) or in dorsoventral view (j, l). Note the warty surface structure in (b) and a broad striated intercalary band in (e). (g–i) Empty thecae showing visible pores in the periphery of the thecal plate. (k–m) Cells stained with DAPI and viewed with epifluorescence and UV excitation. Note the visible thecal pores in (k) and (m). (n, o) The same cell stained with DAPI and viewed with UV excitation (o) to demonstrate the posterior location of the oval nucleus (blue). Scale bars = 5 μm.
**Figure S2.** *Prorocentrum thermophilum* strain CCMP 1260. Light microscope images of formaldehyde‐preserved cells (a–j). Different cells in lateral view (a, f), in apical view (g, i, j) or in dorsoventral view (b, d, h). Note the warty surface structure in (b), the broad striated intercalary band in (b) and (c). (e–j) Cells stained with DAPI and viewed with epifluorescence and UV excitation. Note the large flagella pore and the smaller accessory pore (g, i, j) and the visible thecal pores (f, g, i, j). Scale bars = 5 μm.
**Figure S3.** *Prorocentrum thermophilum* strain CCMP 1787. SEM of different thecae; (a) cell in right lateral view (b) cell in left‐thecal view. (c) Cell in left apical view. (d) Cell in apical view. (e) Interior view of the right thecal plate indicating the position of small (black arrows) and large (white arrows) thecal pores. (f, g) Detailed apical ventral view indicating the row of large pores (white arrows) on the ventral side of the right plate. (h) Detailed right‐lateral view of the periflagellar area. (i, j) Details of the surface ornamentation showing the three‐dimensional structure of the short knob‐like spines and the radial extensions connecting the base of these short projections. Scale bars = 2 μm (a–e) or 1 μm (f–j).
**Figure S4.** *Prorocentrum thermophilum* strain CCMP 1787. SEM of different thecae. (a–e) Apical view of the periflagellar area showing the detailed shape and arrangement of the large flagellar pore (fp), small accessory pore (ap), periflagellar platelets and apical projections. (f) Schematic drawing of the periflagellar area; numbers indicate denominations of the platelets; ap, accessory pore; fp, flagellar pore; gray shows the location of the flat wings on the platelets. Scale bars = 0.5 μm.
**Figure S5.** *Prorocentrum thermophilum* strain CCMP 1260. SEM of different thecae. (a) Cell in right lateral view. (b) Cell in left‐thecal view. (c) Cell in apical view. (d) Detailed right‐lateral view of the periflagellar area. (e) Interior view of the right thecal plate indicating the position of small (black arrows) and large (white arrows) thecal pores. (f) Detailed apical ventral view indicating the row of three large pores (white arrows) on the ventral side of the right plate. (g) Details of the surface ornamentation showing the three‐dimensional structure of the short knob‐like spines and the radial extensions connecting the base of these short projections. (h–j) Apical view of the periflagellar area showing the detailed shape and arrangement of the large flagellar pore (fp), small accessory pore (ap), periflagellar platelets and apical projections; numbers indicate denominations of the platelets; ap, accessory pore; fp, flagellar pore. Scale bars = 2 μm (a–c, e) or 1 μm (d, f–j).
**Figure S6.** *Prorocentrum cordatum* strain 1‐B3. (a, b) Light microscope images of living cells in right lateral view (a), or in dorsoventral view (b). (c–i) SEM of different thecae. (c) Whole cell in right lateral view. (d) Details of the surface ornamentation showing the three‐dimensional structure of the spines and the presence of large (white arrows) and small (black arrows) thecal pores. (e, f) Interior view of thecal plates indicating the position of small (black arrows) and large (white arrows) thecal pores. (g–i) Detailed apical ventral view indicating the row of two large pores (white arrows) on the ventral side of the right thecal plate. Black arrows indicate positions of small pores. Note in (h) the number and arrangement of periflagellar platelets around the accessory pore and flagellar pore. (j) Schematic drawing of the periflagellar area; numbers indicate denominations of the platelets; ap, accessory pore; fp, flagellar pore; gray shows the location of the projections on the platelets, with dark gray highlighting projections dominant in terms of height. Scale bars = 5 μm (a–c, e) or 1 μm (d, f–i).
**Figure S7.** Maximum Likelihood (ML) phylogenetic tree showing alignment of 42 nuclear SSU rRNA gene sequences (1439 bp) demonstrating the sequences from the four *Prorocentrum insidiosum* strains were distinct from all genetically represented species within the genus *Prorocentrum*. The accession number, genus, species and strain code are provided for each sequence. New sequences are shown in bold type and colored boxes represent species delimitations. Species classifications represent current assignment, with the original species classification as it appears in GenBank, also provided. Species type material is indicated by an asterisk. Values at nodes represent ML bootstrap/Bayesian support. Values under 50 and 0.90, respectively, are not shown. The scale bar 0.004 represents substitutions per site.
**Figure S8.** Maximum Likelihood (ML) phylogenetic tree showing alignment of 89 nuclear ITS gene sequences (557 bp) demonstrating the sequences from the four *Prorocentrum insidiosum* strains were distinct from all genetically represented species within the genus *Prorocentrum*. The accession number, genus, species and strain code are provided for each sequence. New sequences are shown in bold type and colored boxes represent species delimitations. Species classifications represent current assignment, with the original species classification as it appears in GenBank, also provided. Species type material is indicated by an asterisk. Values at nodes represent ML bootstrap/Bayesian support. Values under 50 and 0.90 (unless representing a primary lineage), respectively, are not shown. The scale bar 0.2 represents substitutions per site.
**Figure S9.** Maximum Likelihood (ML) phylogenetic tree showing alignment of 60 partial nuclear LSU rRNA gene sequences (623 bp) demonstrating the sequences from the four *Prorocentrum insidiosum* strains were distinct from all genetically represented species within the genus *Prorocentrum*. The accession number, genus, species and strain code are provided for each sequence and colored boxes represent species delimitations. New sequences are shown in bold type. Species classifications represent current assignment, with the original species classification as it appears in GenBank, also provided. Species type material is indicated by an asterisk. Values at nodes represent ML bootstrap/Bayesian support. Values under 50 and 0.90 (unless representing a primary lineage), respectively, are not shown. The scale bar 0.06 represent substitutions per site.
**Table S1.** Details of the sequences included in the SSU + ITS + LSU phylogeny in the order they appear in Figure 7 (main article). Species classifications represent current assignment, with the original species classification as it appears in GenBank, also provided. If “holotype” or “epitype” is noted for a species name, then it refers to material, from which the type was prepared. All names are given under the rules of the ICN, including the author standard forms Brummitt and Powell (1992). Abbreviations: n.inf. = no information, unpubl. = unpublished.

## Data Availability

The data that supports the findings of this study are available in the Supporting Information of this article.
