# Supplementary material for: Prorocentrum insidiosum sp. nov. (Prorocentrales, Dinophyceae): Morphological and Phylogenetic Characterization of a Mucosphere Producing Dinoflagellate From the “cordatum Group”
Source: J Eukaryot Microbiol. 2025 May 30;72(4):e70017. doi: 10.1111/jeu.70017 (PMC12125498; doi:10.1111/jeu.70017)
Supplement: Supplementary file 1 — Figure S1. Prorocentrum thermophilum strain CCMP 1787. Light microscope images of formaldehyde‐preserved cells (a–o). Different cells in lateral view (a–d, g, m, n), in apical view (e, k) or in dorsoventral view (j, l). Note the warty surface structure in (b) and a broad striated intercalary band in (e). (g–i) Empty thecae showing visible pores in the periphery of the thecal plate. (k–m) Cells stained with DAPI and viewed with epifluorescence and UV excitation. Note the visible thecal pores in (k) and (m). (n, o) The same cell stained with DAPI and viewed with UV excitation (o) to demonstrate the posterior location of the oval nucleus (blue). Scale bars = 5 μm. Figure S2. Prorocentrum thermophilum strain CCMP 1260. Light microscope images of formaldehyde‐preserved cells (a–j). Different cells in lateral view (a, f), in apical view (g, i, j) or in dorsoventral view (b, d, h). Note the warty surface structure in (b), the broad striated intercalary band in (b) and (c). (e–j) Cells stained with DAPI and viewed with epifluorescence and UV excitation. Note the large flagella pore and the smaller accessory pore (g, i, j) and the visible thecal pores (f, g, i, j). Scale bars = 5 μm. Figure S3. Prorocentrum thermophilum strain CCMP 1787. SEM of different thecae; (a) cell in right lateral view (b) cell in left‐thecal view. (c) Cell in left apical view. (d) Cell in apical view. (e) Interior view of the right thecal plate indicating the position of small (black arrows) and large (white arrows) thecal pores. (f, g) Detailed apical ventral view indicating the row of large pores (white arrows) on the ventral side of the right plate. (h) Detailed right‐lateral view of the periflagellar area. (i, j) Details of the surface ornamentation showing the three‐dimensional structure of the short knob‐like spines and the radial extensions connecting the base of these short projections. Scale bars = 2 μm (a–e) or 1 μm (f–j). Figure S4. Prorocentrum thermophilum strain CCMP 1787. SEM of differ [file JEU-72-e70017-s001.pdf]

Supplementary Material

***Prorocentrum insidiosum* sp. nov. (Prorocentrales, Dinophyceae): morphological and phylogenetic characterization of the mucosphere producing dinoflagellate from the '*cordatum* group'**

Michaela E. Larsson[1,2]
Gustaaf Hallegraeff[3]
Martina A. Doblin[2,4]
Urban Tillmann[5*]

[1]Aquatic Science Branch, Department of Water and Environmental Regulation, Joondalup, WA 6027, Australia
[2]Climate Change Cluster (C3), University of Technology Sydney, PO Box 123, Broadway, NSW, 2007, Australia
[3]Institute for Marine and Antarctic Studies, University of Tasmania, Private Bag 129, Hobart, TAS, 7001, Australia
[4]Sydney Institute of Marine Science, Mosman, NSW, 2088, Australia
[5]Alfred Wegener Institute. Helmholtz Center for Polar and Marine Research, Am Handelshafen 12, 27570 Bremerhaven, Germany

[*]Corresponding author: urban.tillmann@awi.de, phone 49 471 48311470

1) Additional morphological examination

Prorocentrum thermophilum

For a thorough, morphological comparison of *P. insidiosum* sp. nov. with the closely related *P. thermophilum*, two strains of *P. thermophilum* from the CCMP culture collection (CCMP1787 and CCMP1260) were analysed using light and electron microscopy (Figures S1–S5).

Cells of both CCMP strains of *P. thermophilum* were round in lateral view (Figure S1a–d, Figure S2a, f) and broadly lens-shaped (Figure S1j, S2d) to round in apical/dorsal/ventral view (Figure S1j–l, Figure S2b, h, i, j). In apical/ventral/dorsal view the horizontally striated intercalary band of varying widths was visible (Figures S1e and S2b, c, e). Light microscopy of cells or empty thecae revealed a spiny or warty surface ornamentation and the presence of a few pores located towards the periphery of the cell (Figure S1g, h, m).

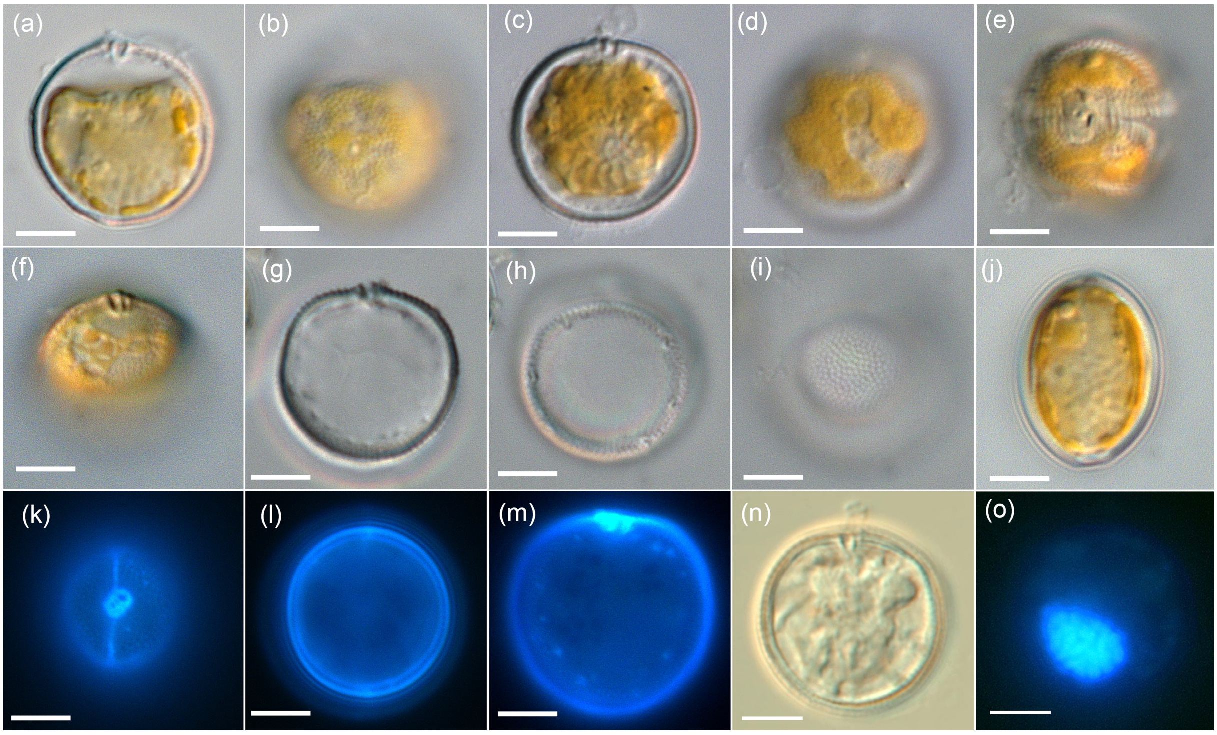

Figure S1: *Prorocentrum thermophilum* strain CCMP 1787. Light microscope images of formaldehyde-preserved cells (a–o). Different cells in lateral view (a–d, g, m, n), in apical view (e, k) or in dorsoventral view (j, l). Note the warty surface structure in (b) and a broad striated intercalary band in (e). (g–i) Empty thecae showing visible pores in the periphery of the thecal plate. (k–m) Cells stained with DAPI and viewed with epifluorescence and UV excitation. Note the visible thecal pores in (k) and (m). (n–o) The same cell stained with DAPI and viewed with UV excitation (o) to demonstrate the posterior location of the oval nucleus (blue). Scale bars = 5 µm.

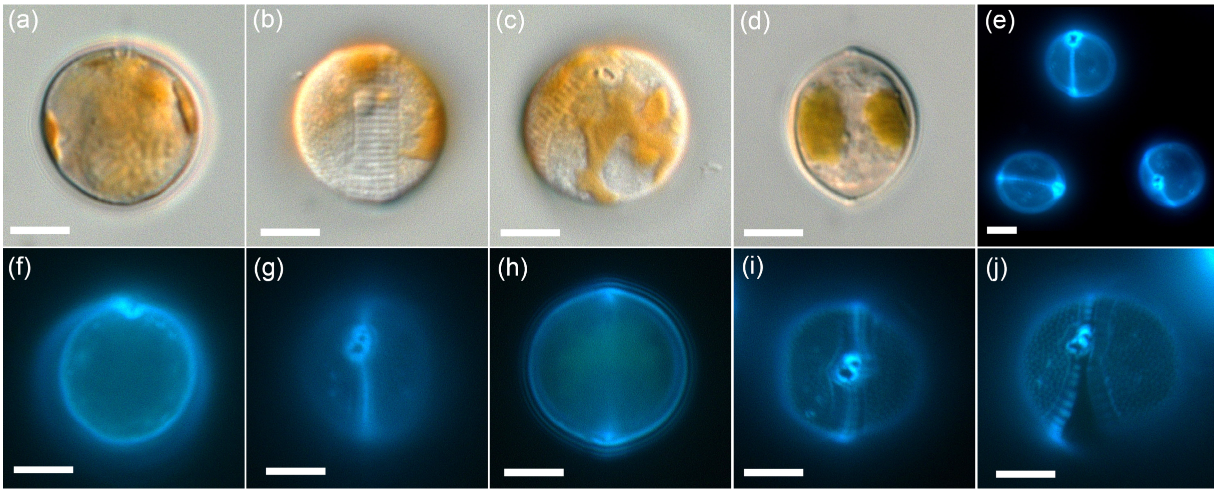

Figure S2: *Prorocentrum thermophilum* strain CCMP 1260. Light microscope images of formaldehyde-preserved cells (a–j). Different cells in lateral view (a, f), in apical view (g, i, j) or in dorsoventral view (b, d, h). Note the warty surface structure in (b), the broad striated intercalary band in (b) and (c). (e–j) Cells stained with DAPI and viewed with epifluorescence and UV excitation. Note the large flagella pore and the smaller accessory pore (g, i, j) and the visible thecal pores (f, g, i, j). Scale bars = 5 µm.

Except for bulging elevations around the two apical pores, there were no prominent apical projections (Figures S1b, c, f, g, m, n and S2a, c, f). An ovoid nucleus was located posteriorly (Figure S1o). Electron microscopy (Figures S3–S5) confirmed the surface ornamentation was formed by short and knob-like spines (Figures S3i, j and S5g) and revealed the presence of two different types of pores, small and large (black and white arrows respectively in Figures S3 and S5). The different pore types were clearly distinguishable in internal plate views (Figures S3e and S5e). There was consistently a row of three large pores on the right thecal plate on the ventral side in apical position (Figures S3f, g and S5f). The flagellar pore area was composed of 8 platelets surrounding a round or oval accessory pore (ap) and a larger round or oval flagellar pore (fp) (Figures S4 and F5h–j). All platelets except the small platelet 4 bearded flat and rim-like projections. Most prominently was the structure on platelet 1, where several of these flat wings were variably arranged mostly parallel and also delineating the accessory pore (Figure S4).

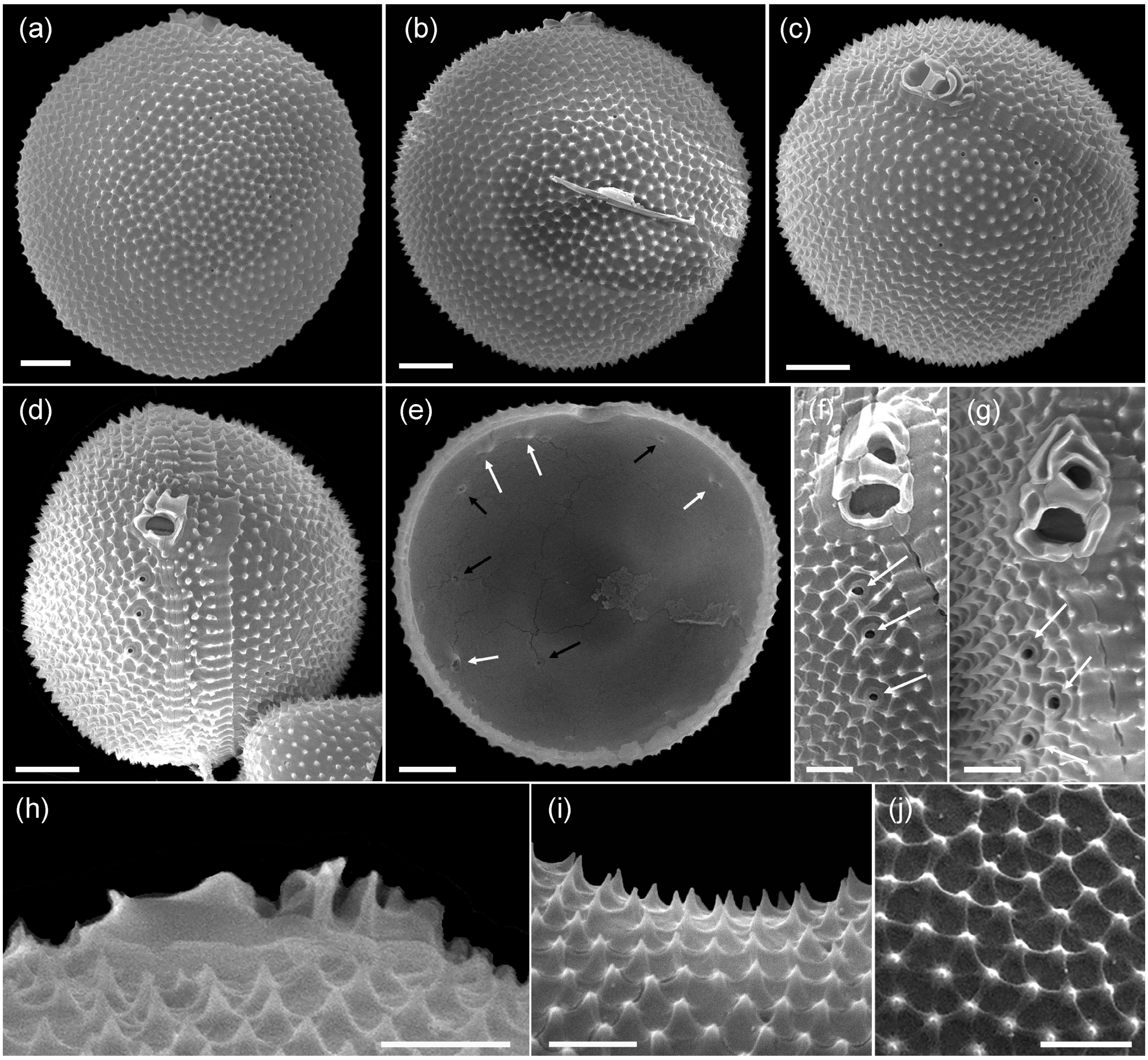

Figure S3: *Prorocentrum thermophilum* strain CCMP 1787. SEM of different thecae; (a) cell in right lateral view (b) cell in left-thecal view. (c) Cell in left apical view. (d) Cell in apical view. (e) Interior view of the right thecal plate indicating the position of small (black arrows) and large (white arrows) thecal pores. (f–g) Detailed apical ventral view indicating the row of large pores (white arrows) on the ventral side of the right plate. (h) Detailed right-lateral view of the periflagellar area. (i–j) Details of the surface ornamentation showing the three-dimensional structure of the short knob-like spines and the radial extensions connecting the base of these short projections. Scale bars = 2 µm (a–e) or 1 µm (f–j).

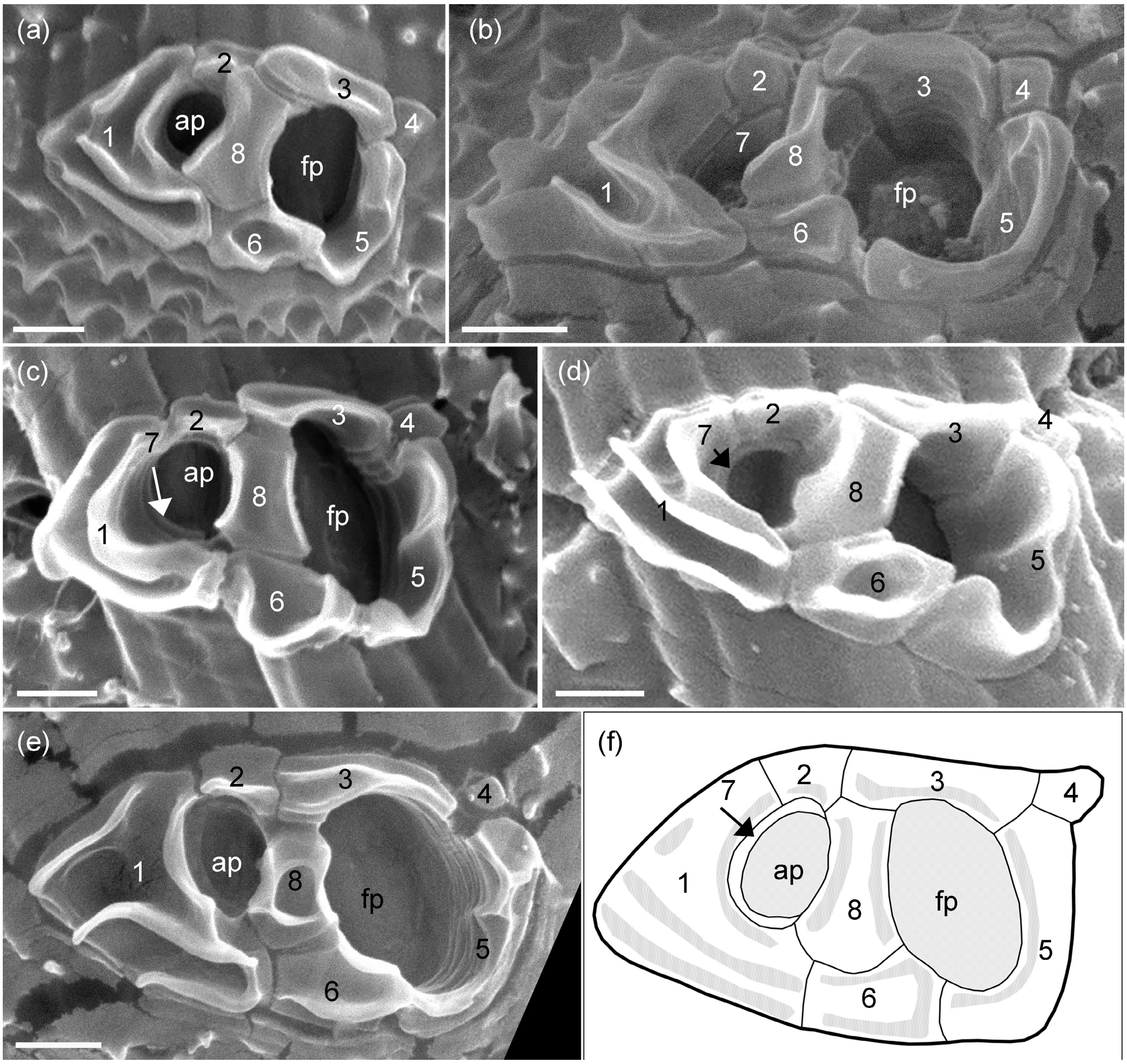

Figure S4: *Prorocentrum thermophilum* strain CCMP 1787. SEM of different thecae. (a–e) Apical view of the periflagellar area showing the detailed shape and arrangement of the large flagellar pore (fp), small accessory pore (ap), periflagellar platelets and apical projections. (f) Schematic drawing of the periflagellar area; numbers indicate denominations of the platelets; ap, accessory pore; fp, flagellar pore; grey shows the location of the flat wings on the platelets. Scale bars = 0.5 µm.

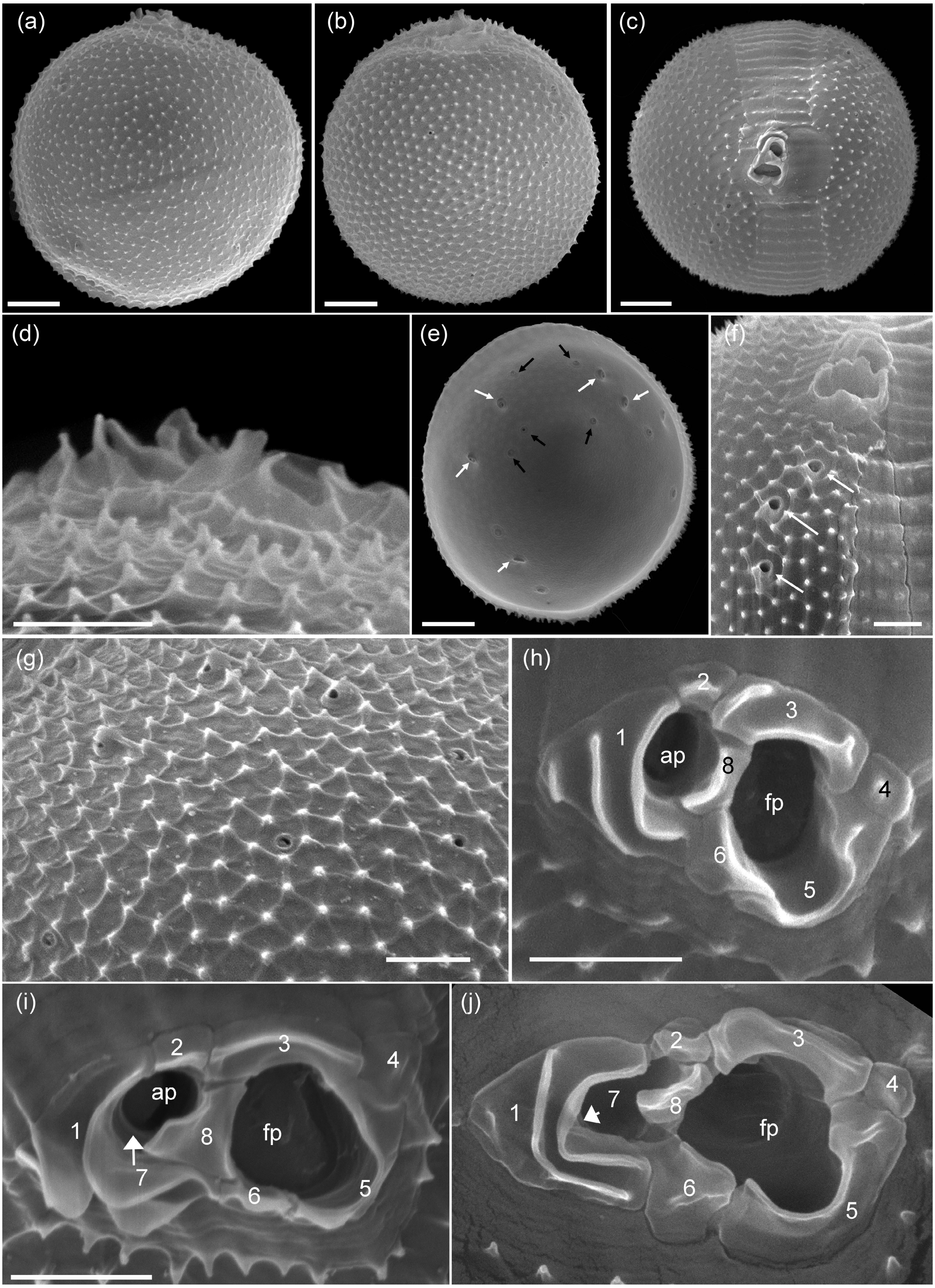

Figure S5: *Prorocentrum thermophilum* strain CCMP 1260. SEM of different thecae. (a) Cell in right lateral view. (b) Cell in left-thecal view. (c) Cell in apical view. (d) Detailed right-lateral view of the periflagellar area. (e) Interior view of the right thecal plate indicating the position of small (black arrows) and large (white arrows) thecal pores. (f) Detailed apical ventral view indicating the row of three large pores (white arrows) on the ventral side of the

right plate. (g) Details of the surface ornamentation showing the three-dimensional structure of the short knob-like spines and the radial extensions connecting the base of these short projections. (h–j) Apical view of the periflagellar area showing the detailed shape and arrangement of the large flagellar pore (fp), small accessory pore (ap), periflagellar platelets and apical projections; numbers indicate denominations of the platelets; ap, accessory pore; fp, flagellar pore. Scale bars = 2 µm (a–c, e) or 1 µm (d, f–j).

Prorocentrum cordatum

Additional morphological observations were performed on one strain of *P. cordatum* (1-B3) isolated from English Channel (50° 14.388' N, 0° 57.366 E) in July 2018 (Figure S6).

Cells of *P. cordatum* strain 1-B3 were oval in lateral view and strongly compresses dorso-ventrally (Figure S6a–b). Cells had a spiny surface with a number of thecal pores located primarily towards the periphery of the thecal plate. There were two different types of pores, small and large (black and white arrows respectively in Figure S6d–i) which were most clearly visible in internal plate views (Figure S6e–f). There were consistently two large pores in a row on the ventral side of the right thecal plate in apical position (Figure S6g–i). There were 8 platelets surrounding the accessory pore (ap) and a larger oval flagellar pore (fp) in the periflagellar area. There were a number of projections on the periflagellar platelets, some of which (e.g. on platelet 6) were higher than wide (i.e. protrusions). The most prominent projection on platelet 1 was composed of two wings in parallel arrangement, whereby the inner part of the wing partially bordered the accessory pore (Figure S6g–i).

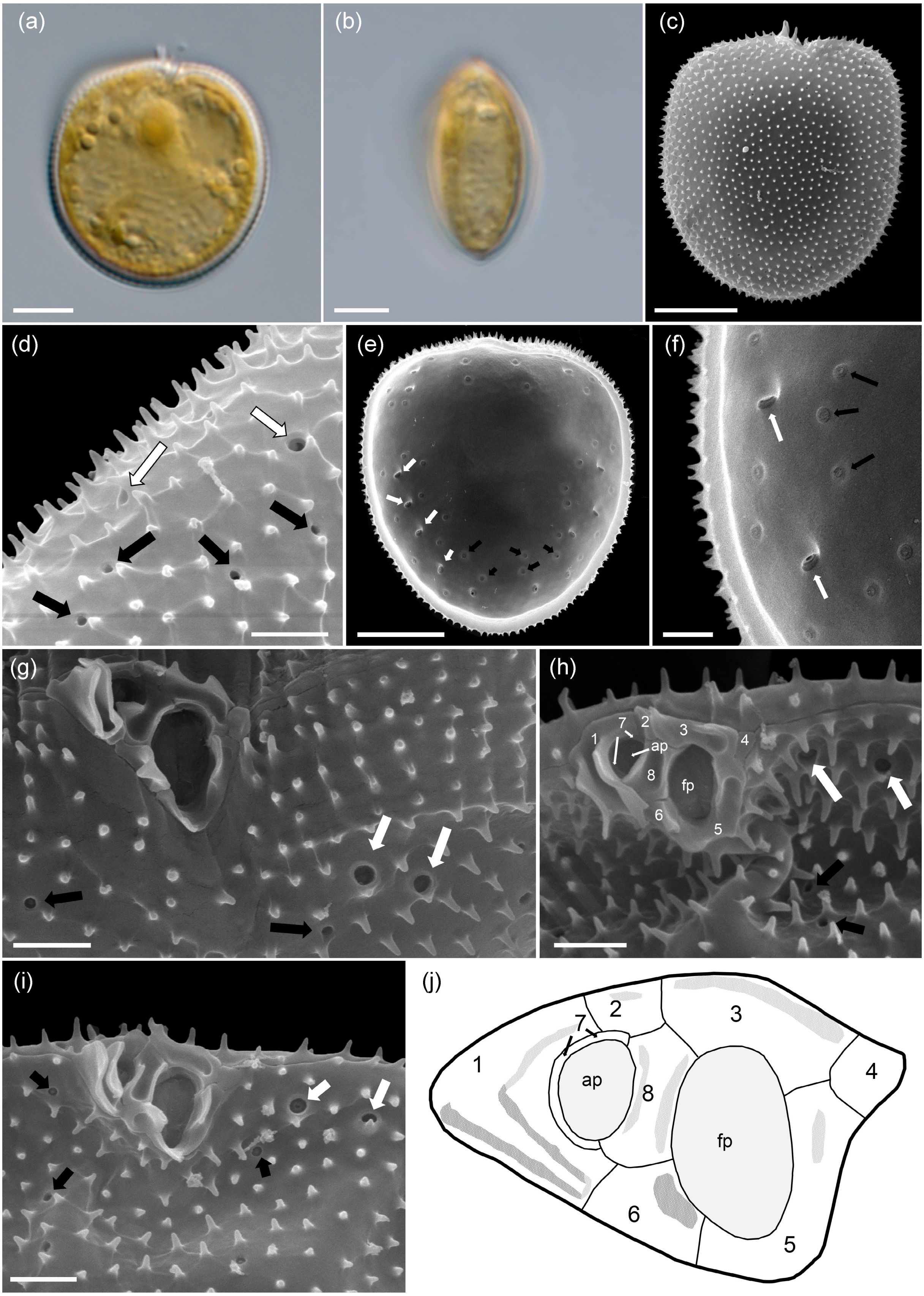

Figure S6: *Prorocentrum cordatum* strain 1-B3. (a–b) Light microscope images of living cells in right lateral view (a), or in dorsoventral view (b). (c–i) SEM of different thecae. (c) Whole cell in right lateral view. (d) Details of the surface ornamentation showing the three-dimensional structure of the spines and the presence of large (white arrows) and small (black arrows) thecal

pores. (e–f) Interior view of thecal plates indicating the position of small (black arrows) and large (white arrows) thecal pores. (g–i) Detailed apical ventral view indicating the row of two large pores (white arrows) on the ventral side of the right thecal plate. Black arrows indicate positions of small pores. Note in (h) the number and arrangement of periflagellar platelets around the accessory pore and flagellar pore. (j) Schematic drawing of the periflagellar area; numbers indicate denominations of the platelets; ap, accessory pore; fp, flagellar pore; grey shows the location of the projections on the platelets, with dark grey highlighting projections dominant in terms of height. Scale bars = 5 µm (a–c, e) or 1 µm (d, f–i).

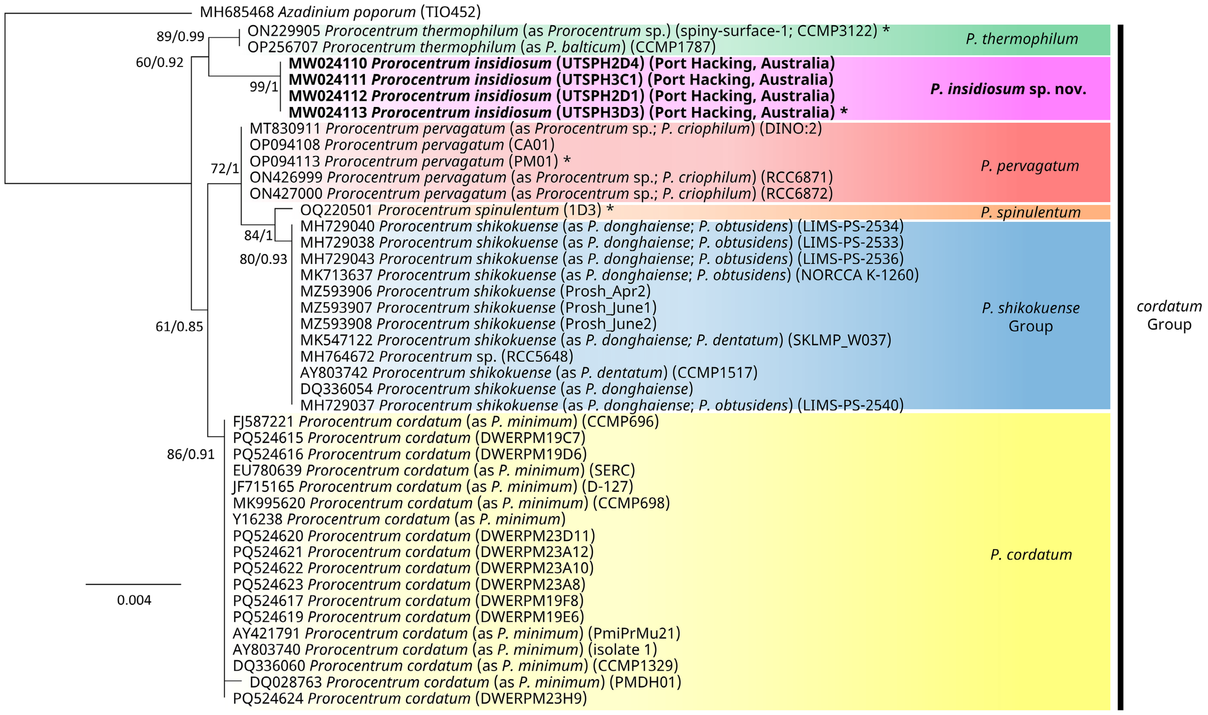

Figure S7: Maximum Likelihood (ML) phylogenetic tree showing alignment of 42 nuclear SSU rRNA gene sequences (1,439 bp) demonstrating the sequences from the four *P. insidiosum* strains were distinct from all genetically represented species within the genus *Prorocentrum*. The accession number, genus, species and strain code are provided for each sequence. New sequences are shown in bold type and colored boxes represent species delimitations. Species classifications represent current assignment, with the original species classification as it appears in GenBank, also provided. Species type material is indicated by an asterisk. Values at nodes represent ML bootstrap/Bayesian support. Values under 50 and 0.90, respectively, are not shown. The scale bar 0.004 represents substitutions per site.

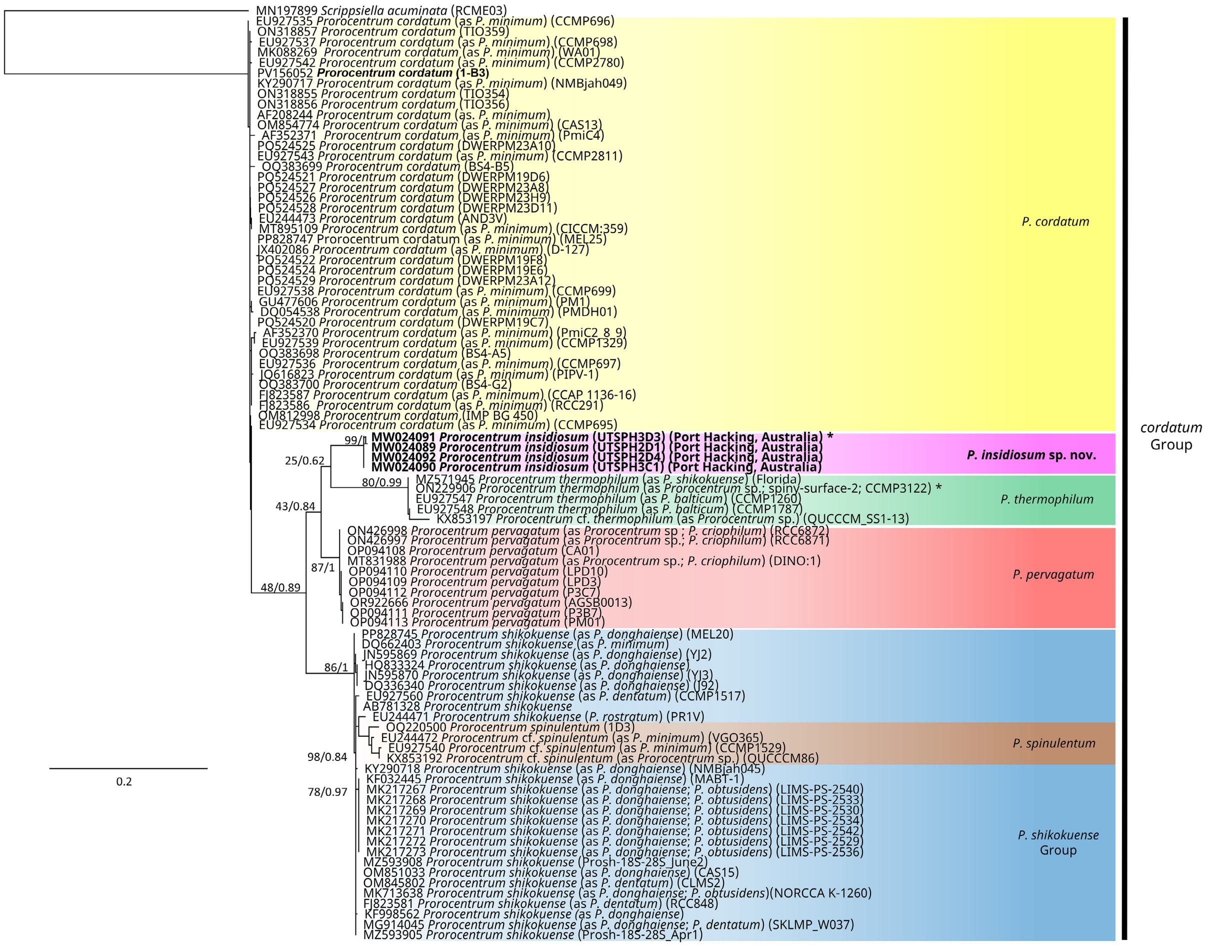

Figure S8: Maximum Likelihood (ML) phylogenetic tree showing alignment of 89 nuclear ITS gene sequences (557 bp) demonstrating the sequences from the four *P. insidiosum* strains were distinct from all genetically represented species within the genus *Prorocentrum*. The accession number, genus, species and strain code are provided for each sequence. New sequences are shown in bold type and colored boxes represent species delimitations. Species classifications represent current assignment, with the original species classification as it appears in GenBank, also provided. Species type material is indicated by an asterisk. Values at nodes represent ML bootstrap/Bayesian support. Values under 50 and 0.90 (unless representing a primary lineage), respectively, are not shown. The scale bar 0.2 represents substitutions per site.

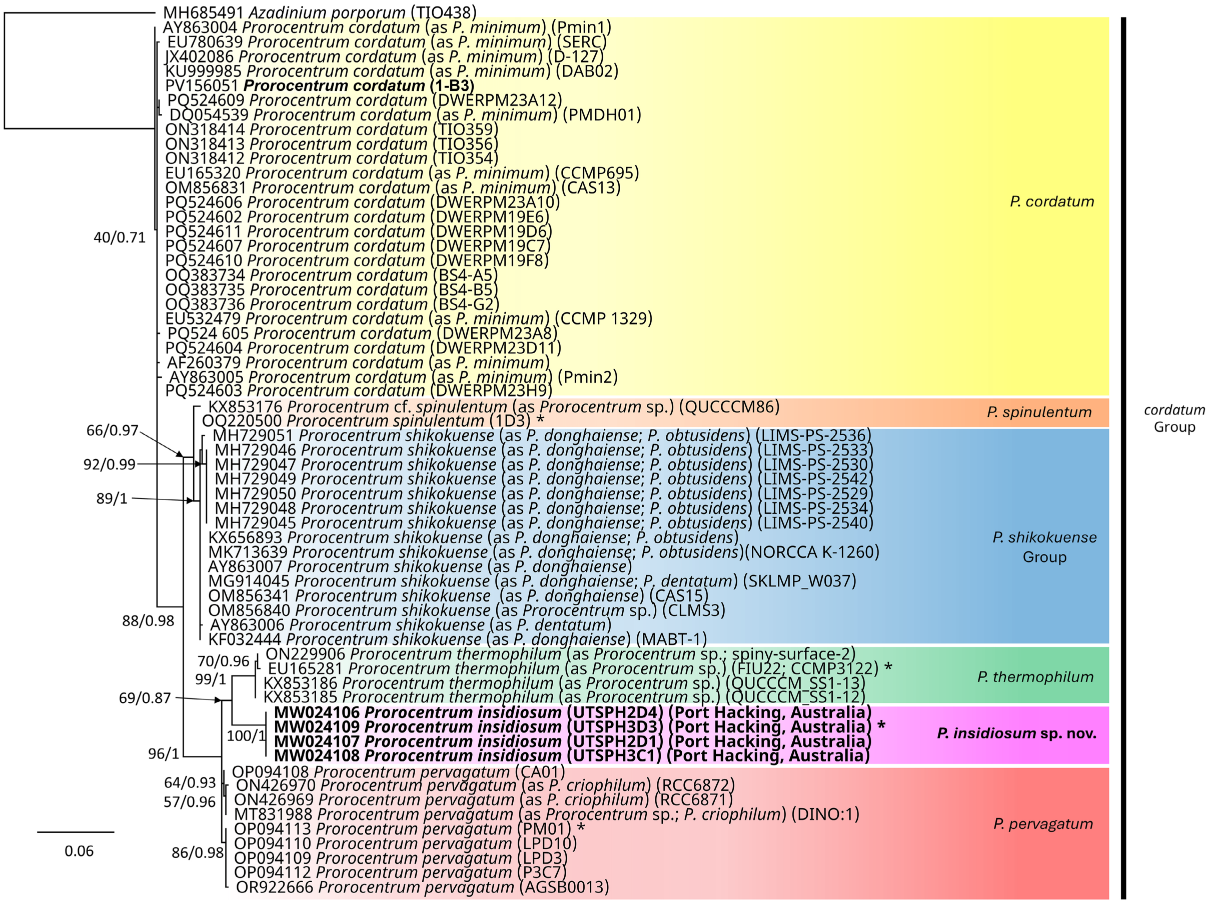

Figure S9: Maximum Likelihood (ML) phylogenetic tree showing alignment of 60 partial nuclear LSU rRNA gene sequences (623 bp) demonstrating the sequences from the four *P. insidiosum* strains were distinct from all genetically represented species within the genus *Prorocentrum*. The accession number, genus, species and strain code are provided for each sequence and colored boxes represent species delimitations. New sequences are shown in bold type. Species classifications represent current assignment, with the original species classification as it appears in GenBank, also provided. Species type material is indicated by an asterisk. Values at nodes represent ML bootstrap/Bayesian support. Values under 50 and 0.90 (unless representing a primary lineage), respectively, are not shown. The scale bar 0.06 represent substitutions per site.

Supplementary Table 1: Details of the sequences included in the SSU+ITS+LSU phylogeny in the order they appear in Figure 7 (main article). Species classifications represent current assignment, with the original species classification as it appears in GenBank, also provided. If 'holotype' or 'epitype' is noted for a species name, then it refers to material, from which the type was prepared. All names are given under the rules of the ICN, including the author standard forms Brummitt and Powell (1992). Abbreviation: n.inf. = no information; unpubl. = unpublished.

Species Name with Authority	Strain No.	Locality	Date	Isolator(s)	GenBank No(s)	Reference
**<u>Outgroup</u>**						
Gymnodinium catenatum H.W.Graham	GnCt01	Eastern Indian Ocean, East China See, off South Korea: Nanpo, Jinhae Bay	n.inf.	n.inf.	DQ785882 (rRNA)	Ki & Han 2007
Prorocentrum foveolatum Croome & P.A.Tyler (reference material)	PFBL01	Australia: Tasmania, Bruny Island, Big Lagoon	Feb, 2002	I. Pearce	AY259173 (LSU)	Pearce & Hallegraeff 2004
Prorocentrum playfairii Croome & P.A.Tyler (reference material)	PPWL01	Australia: Tasmania, Windmill Lagoon	Jan, 2001	I. Pearce	AY259174 (LSU)	Pearce & Hallegraeff 2004
Prorocentrum concavum Fukuyo (holotype of *Prorocentrum arabianum* S.L.Morton & M.A.Faust) (as *Prorocentrum arabianum*)	NCMA1724	Western Indian Ocean, Arabian Sea, Gulf of Oman, off Oman (23°34′N, 58°52'E)	May, 1995	S. Brett	EU927555 (ITS), DQ336184 (LSU)	Ferrell & Beaton (unpubl. 2008), Murray et al. 2007
Prorocentrum leve M.A.Faust, Kibler, Vandersea, P.A.Tester & Litaker (holotype)	NCMA2634	Western North Atlantic, Caribbean Sea, off Belize: Stann Creek, Twin Cays (16°50N, 88°06'W)	May 21, 2002	S. Kibler	DQ238043 (SSU+ITS+LSU)	Faust et al. 2008

Prorocentrum hoffmannianum M.A.Faust	NCMA683 (≡ PL200A)	Western North Atlantic, off USA–FL: Knight Key (24°42'N, 81°08'W)	Nov 1, 1985	J. Bomber	KF885225 (SSU+ITS+LSU), KC622310 (SSU+ITS+LSU)	Lee (unpubl. 2013), Herrera Sepúlveda et al. 2015
Prorocentrum lima (Ehrenb.) F.Stein	NCMA1370 (≡ FIT#69)	Western North Atlantic, off USA–FL: Knight Key (24°42'N, 81°08'W)	n.inf.	J. Bomber	EU927507 (ITS), DQ336180 (LSU)	Ferrell & Beaton (unpubl.) 2008, Murray et al. 2009
Prorocentrum lima (Ehrenb.) F.Stein	NCMA685 (≡ PL2V)	Eastern North Atlantic, off Spain: Galicia, Ría de Vigo (42°14'N, 8°48'W)	n.inf.	I. Bravo	AB189765 (ITS), DQ336179 (LSU)	Ferrell & Beaton (unpubl. 2008), Murray et al. 2009, Nagahama et al. 2011
Prorocentrum lima (Ehrenb.) F.Stein (epitype of *Cryptomonas lima* Ehrenb. and *Exuviaella marina* Cienk.)	NCMA1743 (≡ PA)	Atlantic Ocean, off Canada: Nova Scotia, Lunenburg, Mahone Bay (44°30'N, 64°10'W)	n.inf.	C. Brown & N. Lewis	AB189767 (ITS)	Ferrell & Beaton (unpubl. 2008), Nagahama et al. 2011
Prorocentrum tsawwassenense Hoppenrath & B.S.Leander	IFR456	France: Groix Island	Jul 31, 2007	N. Chomérat	JX912182 (LSU)	Hoppenrath et al. 2013
Prorocentrum tsawwassenense Hoppenrath & B.S.Leander	TIO304	Western North Pacific, Yellow Sea, off China: Shangdong, Qingdao (36°03'N, 120°22'E)	Oct 14, 2015	n.inf.	OP764434 (ITS), OP764421 (LSU)	Wu et al. 2022

Prorocentrum tsawwassenense Hoppenrath & B.S.Leander	KW-P.twas1	Kuwait	n.inf.	W. Ismail & J. Larsen	MH669281 (LSU)	Ismail (unpubl. 2018)
Prorocentrum gracile F.Schütt	TIO419	Western North Pacific, South China Sea, Daya Bay, off China: Guangdong, Shenzhen (22°34'N, 114°36E)	Nov 11, 2016	n.inf.	ON318854 (ITS), ON318411 (LSU)	Pei et al. 2022
Prorocentrum micans Ehrenb. (epitype)	A10	Baltic Sea, off Germany: Schleswig-Holstein, Kiel (54°21'N, 10°09'E)	Oct 20, 2017	U. Tillmann	MK405477 (rRNA)	Tillmann et al. 2019
Prorocentrum micans Ehrenb.	NCMA1589 (≡ 992M3)	USA–RI: Narragansett Bay (41°36'N, 71°24'W)	Sep 19, 1992	P. Hargraves	EU780638 (SSU+ITS+LSU)	Handy et al. 2009
Prorocentrum koreanum M.S.Han, S.Y.Cho & P.B.Wang (holotype)	LMBEV9	Eastern Indian Ocean, East China See, off South Korea: Jangmok	n.inf.	n.inf.	KP711350 (SSU), KP711351 (ITS), KP711352 (LSU)	Han et al. 2016
Prorocentrum koreanum M.S.Han, S.Y.Cho & P.B.Wang	BGERL02	Western North Pacific, South China Sea, Beibu Gulf, off China	n.inf.	Y. Xu	MW979822 (SSU), MW999295 (ITS), MW979767(LSU)	Xu (unpubl. 2021)
Prorocentrum rhathymum A.R.Loebl., Sherley & R.J.Schmidt	NMN16	Western North Pacific, South China Sea, off Malaysia: Sabah	n.inf.	n.inf.	FJ842096 (SSU), FJ155840 (ITS), EF566745 (LSU)	Mohammad-Noor et al. 2007, Caillaud et al. 2010
Prorocentrum rhathymum A.R.Loebl., Sherley & R.J.Schmidt	PRJJ1	Western North Pacific, East China Sea, off Republic of South Korea: Jeju (33°52'N, 126°54'E)	Feb, 2009	n.inf.	HF565181 (SSU+ITS+LSU)	Lim et al. 2013

Prorocentrum triestinum J.Schiller (epitype)	1069	Mediterranean Sea, Adriatic Sea, off Italy: Friuli-Venezia Giulia, Trieste, Santa Croce (45°44'N, 13°41'E)	Sep 19, 2018	A. Beran	MW784603 (SSU), MW784569 (ITS), MW784608 (LSU)	Tillmann et al. 2021
Prorocentrum redfieldii Bursa	BGERL44	Western North Pacific, South China Sea, Beibu Gulf, off China	n.inf.	Y. Xu	MW979864(SSU), MW999337 (ITS), MW979809 (LSU)	Xu (unpubl. 2021)
Prorocentrum cordatum (Ostenf.) J.D.Dodge	DWERPM23A10	Eastern Indian Ocean, Australia: Wilson Inlet, Western Australia	Oct 18, 2021	M. Larsson	PQ524622 (SSU), PQ524525 (ITS), PQ524606 (LSU)	Larsson (unpublished 2024)
Prorocentrum cordatum (Ostenf.) J.D.Dodge	DWERPM23H9	Eastern Indian Ocean, Australia: Wilson Inlet, Western Australia	Oct 18, 2021	M. Larsson	PQ524624 (SSU), PQ524526 (ITS), PQ524603 (LSU)	Tillmann et al. 2023b<br>Larsson (unpublished 2024)
Prorocentrum cordatum (Ostenf.) J.D.Dodge	SERC	n.inf.	n.inf.	n.inf.	EU780639 (SSU+ITS+LSU)	Handy et al. 2009
Prorocentrum cordatum (Ostenf.) J.D.Dodge (as *Prorocentrum minimum*)	CCMP1329 (≡ CCCM541, EXUV)	Western North Atlantic, off USA–NY: New York, Long Island, Great South Bay (40°40'N, 73°15'W)	Jul 1, 1958	I. Pinter	DQ336060 (SSU), EU927539 (ITS), EU532479 (LSU)	Ferrell & Beaton (unpubl. 2008), (Lin et al. 2006, Howard et al. 2009)
Prorocentrum cordatum (Ostenf.) J.D.Dodge	BS4-G2	Southwestern Black Sea	2021	U. Tillmann	OQ383700 (ITS), OQ383736 (LSU)	Tillmann et al. 2023b

Prorocentrum cordatum (Ostenf.) J.D.Dodge	CCAP 1136-16	UK–Scotland: Argyl, Loch Etive (56°17'N, 5°05'W)	2003	Ch. Campbell	MK541783 (SSU), FJ823587 (ITS)	Stern et al. 2012, Rad-Menendez (unpubl. 2019)
Prorocentrum cordatum (Ostenf.) J.D.Dodge	BS4-A5	Southwestern Black Sea	2021	U. Tillmann	OQ383698 (ITS), OQ383734 (LSU)	Tillmann et al. 2023b
Prorocentrum cordatum (Ostenf.) J.D.Dodge (as *Prorocentrum minimum*)	D127	Eastern Indian Ocean, off South Korea: Tongyeong	n.inf.	n.inf.	JX402086 (rRNA)	Cheon & Ki (unpubl. 2013)
Prorocentrum cordatum (Ostenf.) J.D.Dodge	BS4-B5	Southwestern Black Sea	2021	U. Tillmann	OQ383699 (ITS), OQ383735 (LSU)	Tillmann et al. 2023b
Prorocentrum cordatum (Ostenf.) J.D.Dodge	DWERPM19E6	Eastern Indian Ocean, Australia: Murray River, Western Australia	Oct 18, 2021	M. Larsson	PQ524619 (SSU), PQ524524 (ITS), PQ524602 (LSU)	Tillmann et al. 2023b Larsson (unpubl. 2024)
Prorocentrum cordatum (Ostenf.) J.D.Dodge	CAS13	n.inf.	n.inf.	n.inf.	OM854774 (ITS), OM856831 (LSU)	Li (unpubl. 2022)
Prorocentrum cordatum (Ostenf.) J.D.Dodge (as *Prorocentrum minimum*)	PIPV1	Eastern North Pacific, Gulf of California, off Mexico: Baja California, Bahía de La Paz	2000	L. Morquecho	JQ616823 (SSU+ITS+LSU), JQ616845 (LSU)	Herrera Sepúlveda et al. 2013
Prorocentrum cordatum (Ostenf.) J.D.Dodge	DWERPM23A8	Eastern Indian Ocean, Australia: Wilson Inlet, Western Australia	Oct 18, 2021	M. Larsson	PQ524623 (SSU), PQ542527 (ITS), PQ524605 (LSU)	Tillmann et al. 2023b Larsson (unpubl. 2024)

Prorocentrum cordatum (Ostenf.) J.D.Dodge	CCMP698 (≡NCMA30)	western North Atlantic, Maquiot Bay, off USA–ME: Cumberland, Brunswick (43°55'N, 69°56'W)	Dec 1, 1988	R. Selvin	MK995620 (SSU), EU927537 (ITS)	Ferrell and Beaton (unpubl. 2008); Wang (unpubl. 2019)
Prorocentrum cordatum (Ostenf.) J.D.Dodge	CCMP696 (≡1PM)	western North Atlantic, off USA–NY: New York, Long Island, South Shore, East Massapequa (40°40'N, 73°26'W)	Jun 1, 1985	R. Selvin	DQ336072 (SSU), EU927535 (ITS)	Lin et al. 2006, Ferrell and Beaton (unpubl. 2008)
Prorocentrum cordatum (Ostenf.) J.D.Dodge	DWERPM19D6	Eastern Indian Ocean, Australia: Murray River, Western Australia	Oct 18, 2021	M. Larsson	PQ524616 (SSU), PQ524521 (ITS), PQ524611 (LSU)	Larsson (unpubl. 2024)
Prorocentrum cordatum (Ostenf.) J.D.Dodge	DWERPM19C7	Eastern Indian Ocean, Australia: Murray River, Western Australia	Oct 18, 2021	M. Larsson	PQ524615 (SSU), PQ524520 (ITS), PQ524607 (LSU)	Larsson (unpubl. 2024)
Prorocentrum cordatum (Ostenf.) J.D.Dodge	DWERPM23D11	Eastern Indian Ocean, Australia: Wilson Inlet, Western Australia	Oct 18, 2021	M. Larsson	PQ524620 (SSU), PQ524528 (ITS), PQ524604 (LSU)	Larsson (unpubl. 2024)
Prorocentrum cordatum (Ostenf.) J.D.Dodge	DWERPM19F8	Eastern Indian Ocean, Australia: Murray River, Western Australia	Oct 18, 2021	M. Larsson	PQ524617 (SSU), PQ524522 (ITS), PQ524610 (LSU)	Tillmann et al. 2023b Larsson (unpubl. 2024)
Prorocentrum cordatum (Ostenf.) J.D.Dodge	DWERPM23A12	Eastern Indian Ocean, Australia: Wilson Inlet, Western Australia	Oct 18, 2021	M. Larsson	PQ524621 (SSU), PQ524529 (ITS), PQ524609 (LSU)	Larsson (unpubl. 2024)

Prorocentrum cordatum (Ostenf.) J.D.Dodge (as *Prorocentrum minimum*)	PMDH01	Western North Pacific, East China Sea, off China: Fujian	n.inf.	n.inf.	DQ028763 (SSU), DQ054538 (ITS), DQ054539 (LSU)	Hou et al. (unpubl. 2005)
Prorocentrum cordatum (Ostenf.) J.D.Dodge	TIO348	Northwest Pacific Ocean, China: Dalian, Liaoning	n.inf.	n.inf.	ON318858 (ITS), ON618415 (LSU)	Pei et al. (unpubl. 2022)
Prorocentrum cordatum (Ostenf.) J.D.Dodge	TIO354	Northwest Pacific Ocean, China: Dalian, Liaoning	n.inf.	n.inf.	ON318855 (ITS), ON318412 (LSU)	Pei et al. (unpubl. 2022)
Prorocentrum cordatum (Ostenf.) J.D.Dodge	TIO356	Northwest Pacific Ocean, China: Dalian, Liaoning	n.inf.	n.inf.	ON318856 (ITS), ON318413 (LSU)	Pei et al. (unpubl. 2022)
Prorocentrum cordatum (Ostenf.) J.D.Dodge	TIO359	Northwest Pacific Ocean, China: Dalian, Liaoning	n.inf.	n.inf.	ON318857 (ITS), ON318414 (LSU)	Pei et al. (unpubl. 2022)
Prorocentrum pervagatum Tillmann, Hoppenrath & Gottschling (as *Prorocentrum* sp.; *Prorocentrum criophilum*)	DINO:1	Western South Atlantic Antarctica: Ross Sea	summer 2017	P. Rose	MT831988 (ITS+LSU)	Bolinesi et al. 2020, Gómez et al. 2023
Prorocentrum pervagatum Tillmann, Hoppenrath & Gottschling	CA01	Southern Ocean, Potter Cove, off UK: South Shetland Islands, King George Island (62°14'S, 58°42'W)	Jan, 2014	U. Tillmann	OP094108 (SSU+ITS+LSU)	Tillmann et al. 2022
Prorocentrum pervagatum Tillmann, Hoppenrath & Gottschling	LPD10	North Sea, off Denmark (56°38'N, 6°41'E, –3m)	Jun, 2020	U. Tillmann	OP094110 (ITS+LSU)	Tillmann et al. 2022

Prorocentrum pervagatum Tillmann, Hoppenrath & Gottschling	LPD3	North Sea, off Denmark (56°38'N, 6°41'E, –3m)	Jun, 2020	U. Tillmann	OP094109 (ITS+LSU)	Tillmann et al. 2022
Prorocentrum pervagatum Tillmann, Hoppenrath & Gottschling	P3C7	North Sea, off Norway: Utsira (59°19'N, 4°58'E, –3m)	Jul, 2015	U. Tillmann	OP094112 (ITS+LSU)	Tillmann et al. 2022
Prorocentrum pervagatum Tillmann, Hoppenrath & Gottschling (holotype)	PM01	Western North Atlantic, Labrador Sea (56°50'N, 52°13'W, –10m)	Jun 28, 2017	U. Tillmann	OP094113 (SSU+ITS+LSU)	Tillmann et al. 2022
Prorocentrum cf. *thermophilum* F.Gómez, Tangcheng Li, Hu.Zhang & Senjie Lin	QUCCCMSS1-13	Qatar: Arabian Gulf	2013	n.inf.	KX853197 (ITS), KX853186 (LSU)	Al Muftah et al. 2016
Prorocentrum thermophilum F.Gómez, Tangcheng Li, Hu.Zhang & Senjie Lin (as *Prorocentrum balticum*)	CCMP1260 (≡ IVC3, IVC3AX)	Western North Atlantic, Gulf of Mexico	Feb 1, 1981	L. Pavasoli	OP256708 (SSU), EU927547 (ITS), OP231463 (LSU)	Ferrell & Beaton (unpubl. 2008), Gómez et al. 2023
Prorocentrum thermophilum F.Gómez, Tangcheng Li, Hu.Zhang & Senjie Lin (as *Prorocentrum shikokuense*)	Florida	n.inf.	n.inf.	n.inf.	MZ571946 (SSU) MZ571945 (ITS),	Li et al. (unpubl. 2021)

Prorocentrum thermophilum F.Gómez, Tangcheng Li, Hu.Zhang & Senjie Lin (as *Prorocentrum* sp.) (holotype)	CCMP3122	Northwest Atlantic Ocean, Gulf of Mexico, USA: New Pass Bridge, Sarasota, Florida.	n. inf.	J. Winshell	ON229905 (SSU), ON229906 (ITS), EU165281 (LSU)	Gómez et al. 2023
Prorocentrum insidiosum Tillmann, Larsson & Hallegraeff, sp. nov. (as *Prorocentrum* cf. *balticum*)	UTSPH3C1	Western South Pacific, Tasman Sea, off Australia: NSW, Sydney, Port Hacking (34°07'S, 151°13'E)	Sep, 2018	M. Larsson	MW024111 (SSU), MW024090 (ITS), MW024108 (LSU)	Larsson et al. 2022
Prorocentrum insidiosum Tillmann, Larsson & Hallegraeff, sp. nov. (as *Prorocentrum* cf. *balticum*)	UTSPH3D3	Western South Pacific, Tasman Sea, off Australia: NSW, Sydney, Port Hacking (34°07'S, 151°13'E)	Sep, 2018	M. Larsson	MW024113 (SSU), MW024091 (ITS), MW024109 (LSU)	Larsson et al. 2022
Prorocentrum insidiosum Tillmann, Larsson & Hallegraeff, sp. nov. (as *Prorocentrum* cf. *balticum*)	UTSPH2D1	Western South Pacific, Tasman Sea, off Australia: NSW, Sydney, Port Hacking (34°07'S, 151°13'E)	Sep, 2018	M. Larsson	MW024112 (SSU), MW024089 (ITS), MW024107 (LSU)	Larsson et al. 2022
Prorocentrum insidiosum Tillmann, Larsson & Hallegraeff, sp. nov. (as *Prorocentrum* cf. *balticum*)	UTSPH2D4	Western South Pacific, Tasman Sea, off Australia: NSW, Sydney, Port Hacking (34°07'S, 151°13'E)	Sep, 2018	M. Larsson	MW024110 (SSU), MW024092 (ITS), MW024106 (LSU)	Larsson et al. 2022

Prorocentrum dentatum F.Stein 1883	CCMP1517	Eastern South Pacific, off Ecuador (2°32'S, 84°12'W)	May 7, 1991	L. Polans	AY803742 (SSU), EU927560 (ITS), AY833515 (LSU)	Ferrell & Beaton (unpubl. 2008), Lin et al. 2006, Chen et al. 2009
Prorocentrum sp.	RCC922 (≡ Biosope_182_FL1-1)	Eastern South Pacific, off Chile (33°21'S, 78°06'W)	Dec 4, 2004	L. Garczarek & D. Marie	FJ823585 (ITS)	Stern et al. 2012
Prorocentrum rostratum F.Stein	PR1V	n.inf.	n.inf.	n.inf.	EU244471 (ITS)	Rial et al. (unpubl. 2007)
Prorocentrum spinulentum Tillmann, Gottschling & Hoppenrath (holotype)	1D3	Eastern North Atlantic, Celtic Sea, off Ireland (51°01'N, 9°04'W)	Jul 26, 2018	U. Tillmann	OQ220501 (SSU), OQ220500 (ITS+LSU)	Tillmann et al. 2023a
Prorocentrum cf. *spinulentum* Tillmann, Gottschling & Hoppenrath (as *Prorocentrum* sp.)	VGO365	Eastern North Atlantic, off Spain: Galicia, Ría de Vigo	Oct 10, 2010	n.inf.	EU244472 (ITS)	Rial et al. (unpubl. 2007), Tillmann et al. 2023a
Prorocentrum cf. *spinulentum* Tillmann, Gottschling & Hoppenrath	CCMP1529	Eastern South Pacific, off Ecuador (2°40'S, 82°43'W)	Sep 8, 1992	L. Polans	EU927540 (ITS)	Ferrell & Beaton (unpubl. 2008), Tillmann et al. 2023a
Prorocentrum cf. *spinulentum* Tillmann, Gottschling & Hoppenrath (as *Prorocentrum* sp.)	QUCCCM86	Qatar: Arabian Gulf	2013	n.inf.	KX853192 (ITS), KX853176 (LSU)	Al Muftah et al. 2016, Tillmann et al. 2023a

Prorocentrum shikokuense Hada	Prosh-18S-28S_Apr1	Mediterranean Sea, Adriatic Sea, off Italy: Apulia, Brandisi (40°39'N, 17°59'E)	Sep 20, 2018	n.inf.	MZ593905 (SSU+ITS+LSU)	Gómez et al. 2021
Prorocentrum shikokuense Hada	Prosh-18S-28S_June2	Mediterranean Sea, Adriatic Sea, off Italy: Apulia, Brandisi (40°39'N, 17°59'E)	Sep 20, 2018	n.inf.	MZ593908 (SSU+ITS+LSU)	Gómez et al. 2021
Prorocentrum shikokuense Hada	SKLMP_W037	Western North Pacific, South China Sea, off Hong Kong: Nam Fung Chau, Sai Kung, Tai She Wan	Feb, 2015	T.-C. Wai	MG914045 (ITS+LSU)	Leung et al. (unpubl. 2018)
Prorocentrum shikokuense Hada	Prosh-18S-28S_Apr2	Mediterranean Sea, Adriatic Sea, off Italy: Apulia, Brandisi (40°39'N, 17°59'E)	Sep 20, 2018	n.inf.	MZ593906 (SSU+ITS+LSU)	Gómez et al. 2021
Prorocentrum shikokuense Hada	Prosh-18S-28S_June1	Mediterranean Sea, Adriatic Sea, off Italy: Apulia, Brandisi (40°39'N, 17°59'E)	Sep 20, 2018	n.inf.	MZ593907 (SSU+ITS+LSU)	Gómez et al. 2021
Prorocentrum shikokuense (as *Prorocentrum donghaiense*)	CAS15	n.inf.	n.inf.	n.inf.	OM851033 (ITS), OM846341 (LSU)	Li (unpubl. 2022)
Prorocentrum shikokuense (as *Prorocentrum donghaiense; Prorocentrum obtusidens*)	NORCCA K-1260	Eastern North Atlantic, off Spain: Canary Islands, La Gomera, San Sebastián	2009	G. Hansen	MK713637 (SSU), MK713638 (ITS), MK713639 (LSU)	Shin et al. 2019

Prorocentrum shikokuense (as *Prorocentrum dentatum*)	RCC848	Eastern South Pacific (32°24'S, 86°47'W, –20m)	Dec 1, 2004	L. Garczarek & D. Marie	EU106736 (SSU), EU927559 (ITS)	Ferrell & Beaton (unpubl. 2008), Gall et al. 2008
Prorocentrum shikokuense (as *Prorocentrum donghaiense*)	MABT-1	Western North Pacific, East China Sea, off China: Zhejiang	n.inf.	n.inf.	KF032443 (SSU), KF032445 (ITS), KF032444 (LSU)	Chen et al. 2013
Prorocentrum shikokuense (as *Prorocentrum donghaiense; Prorocentrum obtusidens*)	LIMS-PS-2536	Western North Pacific, East China Sea	May, 2017	n.inf.	MH729043 (SSU), MK217273 (ITS), MH729051 (LSU)	Shin et al. 2019
Prorocentrum shikokuense (as *Prorocentrum donghaiense; Prorocentrum obtusidens*)	LIMS-PS-2529	Western North Pacific, East China Sea, off South Korea	May, 2017	n.inf.	MH729042 (SSU), MK217272 (ITS), MH729050 (LSU)	Shin et al. 2019
Prorocentrum shikokuense (as *Prorocentrum donghaiense; Prorocentrum obtusidens*)	LIMS-PS-2542	Western North Pacific, East China Sea, off South Korea	May, 2017	n.inf.	MH729041 (SSU), MK217271 (ITS), MH729049 (LSU)	Shin et al. 2019
Prorocentrum shikokuense (as *Prorocentrum donghaiense; Prorocentrum obtusidens*)	LIMS-PS-2530	Western North Pacific, East China Sea	May, 2017	n.inf.	MH729039 (SSU), MK217269 (ITS), MH729047 (LSU)	Shin et al. 2019
Prorocentrum shikokuense (as *Prorocentrum donghaiense; Prorocentrum obtusidens*)	LIMS-PS-2540	Western North Pacific, East China Sea, Gamak Bay, off South Korea: South Jeolla	May, 2017	n.inf.	MH729037 (SSU), MK217267 (ITS), MH729045 (LSU)	Shin et al. 2019

Prorocentrum shikokuense (as *Prorocentrum donghaiense; Prorocentrum obtusidens*)	LIMS-PS-2534	Western North Pacific, East China Sea	May, 2017	n.inf.	MH729040 (SSU), MK217270 (ITS), MH729048 (LSU)	Shin et al. 2019
Prorocentrum shikokuense (as *Prorocentrum donghaiense; Prorocentrum obtusidens*)	LIMS-PS-2533	Western North Pacific, East China Sea	May, 2017	n.inf.	MH729038 (SSU), MK217268 (ITS), MH729046 (LSU)	Shin et al. 2019

References

Al Muftah, A., Selwood, A. I., Foss, A. J., Al-Jabri, H. M., Potts, M. & Yilmaz, M. 2016. Algal toxins and producers in the marine waters of Qatar, Arabian Gulf. *Toxicon* 122:54-66.

Bolinesi, F., Saggiomo, M., Aceto, S., Cordone, A., Serino, E., Valoroso, M. C. & Mangoni, O. 2020. On the Relationship between a novel *Prorocentrum* sp. and colonial *Phaeocystis antarctic*a under iron and vitamin B-12 limitation: Ecological implications for Antarctic waters. *Applied Sciences-Basel* 10.

Brummitt, R. K. & Powell, C. E. 1992. *Authors of Plant Names: a list of authors of scientific names of plants, with recommended standard forms of their names, including abbrevations*. Royal Botanic Gardens,

Caillaud, A., de la Iglesia, P., Campas, M., Elandaloussi, L., Fernandez, M., Mohammad-Noor, N., Andree, K. & Diogene, J. 2010. Evidence of okadaic acid production in a cultured strain of the marine dinoflagellate *Prorocentrum rhathymum* from Malaysia. *Toxicon* 55:633-37.

Chen, G., Ma, C., Zhang, C., Zhou, J., Wang, Y., Wang, G., Zhang, B., Xu, Z. & Lu, D. D. 2013. A rapid and sensitive method for field detection of *Prorocentrum donghaiense* using reverse transcription-coupled loop-mediated isothermal amplification. *Harmful Algae* 29:31-39.

Chen, J., Zhen, Y., Mi, T. & Yu, Z. 2009. Detection of *Prorocentrum donghaiense* using sandwich hybridization integrated with nuclease protection assay. *Acta Oceanologica Sinica* 28:121-26.

Faust, M. A., Vandersea, M. W., Kibler, S. R., Tester, P. A. & Litaker, R. W. 2008. *Prorocentrum levis*, a new benthic species (Dinophyceae) from a mangrove island, Twin Cays, Belize. *Journal of Phycology* 44:232-40.

Gall, F. L., Rigaut-Jalabert, F., Marie, D., Garczarek, L., Viprey, M., Gobet, A. & Vaulot, D. 2008. Picoplankton diversity in the South-East Pacific Ocean from cultures. *Biogeosciences* 5:203–14.

Gómez, F., Gourvil, P., Li, T., Huang, Y., Zhang, H., Courcot, L., Artigas, L. F., Soler Onís, E., Gutiérrez-Rodríguez, A. & Lin, S. 2023. Molecular phylogeny of the spiny-surfaced species of the dinoflagellate *Prorocentrum* with the description of *P. thermophilum* sp. nov. and *P. criophilum* sp. nov. (Prorocentrales, Dinophyceae). *Journal of Phycology* 59:70-86.

Gómez, F., Zhang, H., Roselli, L. & Lin, S. 2021. Detection of *Prorocentrum shikokuense* in the Mediterranean Sea and evidence that *P. dentatum, P. obtusidens* and *P. shikokuense* are three different species (Prorocentrales, Dinophyceae). *Acta Protozoologica* 60:47-59.

Han, M.-S., Wang, P., Kim, J. H., Cho, S.-Y., Park, B. S., Kim, J.-H., Katano, T. & Kim, B.-H. 2016. Morphological and molecular phylogenetic position of *Prorocentrum micans* sensu stricto and description of *Prorocentrum koreanum* sp. nov. from southern coastal waters in Korea and Japan. *Protist* 167:32-50.

Handy, S. M., Bachvaroff, T. R., Timme, R. E., Coats, D. W., Kim, S. & Delwiche, C. F. 2009. Phylogeny of four Dinophysiacean genera (Dinophyceae, Dinophysiales) based on rDNA sequences from single cells and environmental samples. *Journal of Phycology* 45:1163-74.

Herrera Sepúlveda, A., Hernandez-Saavedra, N. Y., Medlin, L. K. & West, N. 2013. Capillary electrophoresis finger print technique (CE-SSCP): An alternative tool for the monitoring activities of HAB species in Baja California Sur Costal. *Environmental Science and Pollution Research* 20:6863-71.

Herrera Sepúlveda, A., Medlin, L. K., Murugan, G., Sierra-Beltrán, A. P., Cruz-Villacorta, A. A. & Hernández-Saavedra, N. Y. 2015. Are *Prorocentrum hoffmannianum* and *Prorocentrum belizeanum* (Dinophyceae, Prorocentrales), the same species? An integration of morphological and molecular data. *Journal of Phycology* 51:173-88.

Hoppenrath, M., Chomérat, N., Horiguchi, T., Schweikert, M., Nagahama, Y. & Murray, S. 2013. Taxonomy and phylogeny of the benthic *Prorocentrum* species (Dinophyceae)—A proposal and review. *Harmful algae* 27:1-28.

Howard, M. D. A., Smith, G. J. & Kudela, R. M. 2009. Phylogenetic relationships of yessotoxin-producing dinoflagellates, based on the large subunit and Internal Transcribed Spacer ribosomal DNA domains. *Applied and Environmental Microbiology* 75:54-63.

Ki, J.-S. & Han, M.-s. 2007. Informative characteristics of 12 divergent domains in complete large subunit rDNA sequences from the harmful dinoflagellate genus, *Alexandrium* (Dinophyceae). *Journal of Eukaryotic Microbiology* 54:210-19.

Larsson, M. E., Bramucci, A. R., Collins, S., Hallegraeff, G., Kahlke, T., Raina, J. B., Seymour, J. R. & Doblin, M. A. 2022. Mucospheres produced by a mixotrophic protist impact ocean carbon cycling. *Nature Communications* 13.

Lim, A. S., Jeong, H. J., Jang, T. Y., Kang, N. S., Lee, S. Y., Yoo, Y. D. & Kim, H. S. 2013. Morphology and molecular characterization of the epiphytic dinoflagellate *Prorocentrum* cf. *rhathymum* in temperate waters off Jeju Island, Korea. *Ocean Science Journal* 48:1-17.

Lin, S., Zhang, H. & Jiao, N. Z. 2006. Potential utility of mitochondrial cytochrome b and ITS mRNA editing in resolving closely related dinoflagellates: A case study of *Prorocentrum* (Dinophyceae). *Journal of Phycology* 42:646–54.

Mohammad-Noor, N., Moestrup, Ø. & Daugbjerg, N. 2007. Light, electron microscopy and DNA sequences of the dinoflageflate *Prorocentrum concavum* (syn. *P. arabianum*) with special emphasis on the periflagellar area. *Phycologia* 46:549-64.

Murray, S. A., Ip, C. L. C., Moore, R., Nagahama, Y. & Fukuyo, Y. 2009. Are prorocentroid dinoflagellates monophyletic? A study of 25 species based on nuclear and mitochondrial genes. *Protist* 160:245–64.

Murray, S. A., Nagahama, Y. & Fukuyo, Y. 2007. Phylogenetic study of benthic, spine-bearing prorocentroids, including *Prorocentrum fukuyoi* sp. nov. *Phycological Research* 55:91–102.

Nagahama, Y., Murray, S. A., Tomaru, A. & Fukuyo, Y. 2011. Species boundaries in the toxic dinoflagellate *Prorocentrum lima* (Dinophyceae, Prorocentrales), based on morphological and phylogenetic characters. *Journal of Phycology* 47:178-89.

Pearce, I. & Hallegraeff, G. M. 2004. Genetic affinities, ecophysiology and toxicity of *Prorocentrum playfairii* and *P. foveolata* (Dinophyceae) from Tasmanian freshwaters. *Phycologia* 43:271-81.

Pei, L. L., Hu, W. J., Wang, P. B., Kang, J. H., Mohame, H. F., Wang, C. Y., Liu, L. M. & Luo, Z. H. 2022. Morphologic and phylogenic characterization of two bloom-forming planktonic *Prorocentrum* (Dinophyceae) species and their potential distribution in the China Sea. *Algal Research-Biomass Biofuels and Bioproducts* 66.

Shin, H. H., Li, Z., Mertens, K. N., Seo, M. H., Gu, H., Lim, W. A., Yoon, Y. H., Soh, H. Y. & Matsuoka, K. 2019. *Prorocentrum shikokuense* Hada and *P. donghaiense* Lu are junior synonyms of *P. obtusidens* Schiller, but not of *P. dentatum* Stein (Prorocentrales, Dinophyceae). *Harmful algae* 89:101686.

Stern, R. F., Andersen, R. A., Jameson, I., Küpper, F. C., Coffroth, M.-A., Vaulot, D., Le Gall, F., Véron, B., Brand, J. J., Skelton, H., Kasai, F., Lilly, E. L. & Keeling, P. J. 2012.

Evaluating the ribosomal Internal Transcribed Spacer (ITS) as a candidate dinoflagellate barcode marker. *PLoS One* 7:e42780.

Tillmann, U., Beran, A., Gottschling, M., Wietkamp, S. & Hoppenrath, M. 2021. Clarifying confusion–*Prorocentrum triestinum* J. Schiller and *Prorocentrum redfieldii* Bursa (Prorocentrales, Dinophyceae) are two different species. *European Journal of Phycology* 57:207-26.

Tillmann, U., Gottschling, M., Wietkamp, S. & Hoppenrath, M. 2023a. Morphological and phylogenetic characterisation of *Prorocentrum spinulentum*, sp. nov. (Prorocentrales, Dinophyceae), a small spiny species from the North Atlantic. *Microorganisms* 11:271.

Tillmann, U., Hoppenrath, M. & Gottschling, M. 2019. Reliable determination of *Prorocentrum micans* Ehrenb. (Prorocentrales, Dinophyceae) based on newly collected material from the type locality. *European Journal of Phycology* 54:417-31.

Tillmann, U., Mitra, A., Flynn, K. J. & Larsson, M. E. 2023b. Mucus-trap-assisted feeding is a common strategy of the small mixoplanktonic *Prorocentrum pervagatum* and *P. cordatum* (Prorocentrales, Dinophyceae). *Microorganisms* 11:1730.

Tillmann, U., Wietkamp, S., Gottschling, M. & Hoppenrath, M. 2022. *Prorocentrum pervagatum* sp. nov. (Prorocentrales, Dinophyceae): A new, small, planktonic species with a global distribution. *Phycological Research* 71:56-71.

Wu, Y. X., Huang, S. N., Krock, B., Leaw, C. P., Teng, S. T., Piumsomboon, A., Punnarak, P., Roeroe, K. A., Wang, N. & Gu, H. F. 2022. Cryptic speciation of benthic *Prorocentrum* (Dinophyceae) species and their potential as ecological indicators. *Journal of Sea Research* 190.
